# A scoping review on the conduct and reporting of scoping reviews

**DOI:** 10.1186/s12874-016-0116-4

**Published:** 2016-02-09

**Authors:** Andrea C. Tricco, Erin Lillie, Wasifa Zarin, Kelly O’Brien, Heather Colquhoun, Monika Kastner, Danielle Levac, Carmen Ng, Jane Pearson Sharpe, Katherine Wilson, Meghan Kenny, Rachel Warren, Charlotte Wilson, Henry T. Stelfox, Sharon E. Straus

**Affiliations:** Li Ka Shing Knowledge Institute of St. Michael’s Hospital, 209 Victoria Street, Toronto, ON M5B 1 W8 Canada; Epidemiology Division, Dalla Lana School of Public Health, University of Toronto, 6th floor, 155 College St, Toronto, ON M5T 3 M7 Canada; Department of Physical Therapy, University of Toronto, 500 University Avenue, Room 160, Toronto, ON M5G 1 V7 Canada; Institute of Health Policy, Management and Evaluation, University of Toronto, 4th Floor, 155 College St, Toronto, ON M5T 3 M6 Canada; Department of Occupational Science & Occupational Therapy, University of Toronto, 160-500 University Avenue, Toronto, ON M5G 1 V7 Canada; School of Rehabilitation Science, University of Ottawa, 200 Lees Avenue, Room A120, Ottawa, ON K1N 6 N5 Canada; Department of Critical Care Medicine, University of Calgary, 3280 Hospital Drive NW, Calgary, AB T2N 2 T9 Canada; Department of Medicine, Faculty of Medicine, University of Toronto, 27 Kings College Circle, Toronto, ON M5S 1A1 Canada

**Keywords:** Scoping reviews, Reporting, Knowledge synthesis, Systematic review, Methods

## Abstract

**Background:**

Scoping reviews are used to identify knowledge gaps, set research agendas, and identify implications for decision-making. The conduct and reporting of scoping reviews is inconsistent in the literature. We conducted a scoping review to identify: papers that utilized and/or described scoping review methods; guidelines for reporting scoping reviews; and studies that assessed the quality of reporting of scoping reviews.

**Methods:**

We searched nine electronic databases for published and unpublished literature scoping review papers, scoping review methodology, and reporting guidance for scoping reviews. Two independent reviewers screened citations for inclusion. Data abstraction was performed by one reviewer and verified by a second reviewer. Quantitative (e.g. frequencies of methods) and qualitative (i.e. content analysis of the methods) syntheses were conducted.

**Results:**

After searching 1525 citations and 874 full-text papers, 516 articles were included, of which 494 were scoping reviews. The 494 scoping reviews were disseminated between 1999 and 2014, with 45 % published after 2012. Most of the scoping reviews were conducted in North America (53 %) or Europe (38 %), and reported a public source of funding (64 %). The number of studies included in the scoping reviews ranged from 1 to 2600 (mean of 118). Using the Joanna Briggs Institute methodology guidance for scoping reviews, only 13 % of the scoping reviews reported the use of a protocol, 36 % used two reviewers for selecting citations for inclusion, 29 % used two reviewers for full-text screening, 30 % used two reviewers for data charting, and 43 % used a pre-defined charting form. In most cases, the results of the scoping review were used to identify evidence gaps (85 %), provide recommendations for future research (84 %), or identify strengths and limitations (69 %). We did not identify any guidelines for reporting scoping reviews or studies that assessed the quality of scoping review reporting.

**Conclusion:**

The number of scoping reviews conducted per year has steadily increased since 2012. Scoping reviews are used to inform research agendas and identify implications for policy or practice. As such, improvements in reporting and conduct are imperative. Further research on scoping review methodology is warranted, and in particular, there is need for a guideline to standardize reporting.

**Electronic supplementary material:**

The online version of this article (doi:10.1186/s12874-016-0116-4) contains supplementary material, which is available to authorized users.

## Background

Scoping reviews are used to map the concepts underpinning a research area and the main sources and types of evidence available [1]. Although scoping review methods have been proposed by Arksey and O’Malley (2005) [1] and further advanced by Levac et al. (2010) [2] and others [3], there is a lack of consistency in terminology and methods reported [4]. This is problematic because when different methods are applied to the same question, they may produce different results, undermining the utility and confidence in knowledge syntheses [5, 6]. As with other types of knowledge syntheses, it is critical to clarify scoping review methods in order to develop a standard that can be put into practice. To address this, the Joanna Briggs Institute published methodological guidance for the conduct of scoping reviews in 2015 [3]. As this is a very recent publication, the methods of published scoping reviews have not been compared for consistency with the methods guidance from this manual.

Although related [7], scoping reviews differ from systematic reviews in a number of ways. Scoping reviews are used to present a broad overview of the evidence pertaining to a topic, irrespective of study quality, and are useful when examining areas that are emerging, to clarify key concepts and identify gaps [3]. For example, scoping reviews can be used to identify a topic area for a future systematic review. Systematic reviews, on the other hand, are used to address more specific questions, based on particular criteria of interest (i.e. population, intervention, outcome, etc.), defined *a priori* [3]. Scoping reviews can be seen as a hypothesis-generating exercise, while systematic reviews can be hypothesis - testing.

An important component of developing a standard methodology for scoping reviews involves creating reporting guidelines. A reporting guideline is a tool (e.g., checklist) that is developed using explicit methods to guide authors in reporting research [8]. Use of reporting checklists increases transparency of methods, and allows readers to judge validity and reliability and use research appropriately [9, 10]. Currently, a checklist for reporting scoping reviews in the Enhancing the QUAlity and Transparency of health Research (EQUATOR) library does not exist for health research [11].

Given that scoping reviews are being conducted in increasing numbers [12] and the lack of consistency in terminology and methods reported [4], a checklist for reporting is essential. Such a reporting checklist would develop a reporting standard that can be put into practice and will complement the methodological guidance on scoping reviews published by the Joanna Briggs Institute [3]. Our objective was to complete a scoping review within the healthcare context to synthesize: 1) articles that utilized and/or described scoping review methods; 2) guidelines for reporting scoping reviews; and 3) studies that assessed the quality of reporting of scoping reviews.

## Methods

### Protocol

Our protocol was developed using the scoping review methodological framework proposed by Arksey and O’Malley (2005) [1] and further refined by the Joanna Briggs Institute [3]. The draft protocol was revised upon receiving feedback from the research team, including methodologists and healthcare providers, as well as the peer-review panel of the Canadian Institutes of Health Research. The final version of the protocol is available upon request from the corresponding author.

### Eligibility criteria

We included the following types of papers: 1) all scoping reviews that utilized a scoping review approach with a description of the literature synthesis method used; 2) short reports describing development, dissemination, use or comparison of scoping review methods versus other knowledge synthesis methods; 3) guidelines for reporting scoping reviews (which may include a checklist, flow diagram or text to guide authors in scoping review reporting, developed using explicit methods); and, 4) studies assessing the quality of reporting and potential sources of bias in scoping reviews. The definition of a scoping review used was as follows: scoping studies [or scoping reviews] “aim to map rapidly the key concepts underpinning a research area and the main sources and types of evidence available, and can be undertaken as stand-alone projects in their own right, especially where an area is complex or has not been reviewed comprehensively before” [1]. We used the Levac et al. (2010) [2] modifications to the original framework of a scoping review [1] to guide this research. This framework includes the following steps: 1) Identify the research question by clarifying and linking the purpose and research question, 2) identify relevant studies by balancing feasibility with breadth and comprehensiveness, 3) select studies using an iterative team approach to study selection and data extraction, 4) chart the data incorporating numerical summary and qualitative thematic analysis, 5) collate, summarize and report the results, including the implications for policy, practice or research, and 6) consultation exercise, which is an optional step and can be adopted as a required component of a scoping review.

All study designs were eligible, including those that utilized qualitative or quantitative methods, methodology or guideline reports. We focused our inclusion criteria to capture scoping review methods within the domain of health, which was defined using the World Health Organization (WHO) definition as ‘a state of complete physical, mental and social well-being’ [13]. As this definition encompassed the social determinants of health, we included scoping reviews conducted within psychology, education and sociology. We also included the philosophy discipline because some knowledge synthesis methods (such as realist reviews) are rooted in philosophy. We excluded publications that did not synthesize literature; for example, epidemiological or financial/administrative “scoping studies”, which typically complete scoping of surveillance or administrative databases as opposed to conducting a search and synthesis of the literature.

### Information sources and search strategy

Comprehensive literature searches were conducted by an expert information specialist in consultation with the research team. First, we searched the following nine electronic databases from inception until August 24, 2014: MEDLINE, EMBASE, Cumulative Index to Nursing and Allied Health Literature (CINAHL), The Cochrane Library, PsycInfo, Social Science Abstracts, Library and Information Science Abstracts (LISA), Philosopher’s Index, and Education Resources Information Center (ERIC). The search was peer-reviewed by another expert librarian using the Peer Review of Electronic Search Strategies checklist, and modified as required [14]. We also searched for grey literature (i.e. difficult to locate or unpublished material) using the Canadian Agency for Drugs and Technologies in Health approach [15]. Specifically, we searched Google and websites of agencies that fund, report or conduct scoping reviews, including the Canadian Institutes of Health Research, Joanna Briggs Institute, and EQUATOR. The search strategy was not limited by study design, language, or year. We intended to include all languages of dissemination but had to limit to English due to the large number of identified papers. The final search strategy for the MEDLINE database is presented in Additional file 1: Appendix A. Additional search strategies are available from the corresponding author, upon request. We also scanned references of a relevant review [16] and a database of scoping reviews shared through personal communication (provided by Shannon Kelly to Dr. Tricco).

### Study selection process

Search results were imported into our online systematic review software called Synthesi.SR [17]. The inclusion criteria were imported into the software as a questionnaire that was developed *a priori* and were used for screening citations (i.e., titles and abstracts) during level 1 screening, and full-text articles during level 2 screening.

To ensure reliability between reviewers, a series of training exercises was conducted prior to commencing screening. Inter-rater agreement for study inclusion was calculated using percent agreement; when it reached > 75 % across the team, we proceeded to the next stage. If lower agreement was observed, the inclusion and exclusion criteria were clarified and another pilot-test occurred. Three rounds of pilot-tests were required for title and abstract screening on a random sample of 92 citations in total across the three pilot-tests. Subsequently, groups of two reviewers (CN, CW, EL, PR, WZ) screened titles and abstracts for inclusion, independently. For full-text screening, two rounds of pilot tests were employed on a random sample of 50 articles in total. Using the same process, groups of two reviewers (CN, CW, EL, PR, WZ) subsequently screened the full-text of potentially relevant articles to determine inclusion using similar inclusion and exclusion criteria. All discrepancies between reviewers were resolved by a single arbitrator (ACT).

### Data items and data collection process

For included articles that were scoping reviews, we abstracted data on study characteristics (e.g., year of study conduct, funding source), objectives, terminology used, seminal papers to guide the methods, and methodological steps in the conduct of the scoping review (e.g., details on the literature search, screening, data abstraction process). Since the sixth step of a scoping review is a consultation exercise, we also abstracted data on the knowledge translation strategies. For included articles reporting guidelines of scoping reviews, we planned to abstract data based on a checklist for developing reporting guidelines [18], which included five domains: study characteristics, background (evidence on quality of reporting, conduct of review to inform guideline); consensus activities (e.g., was a Delphi exercise conducted); face-to-face meetings (e.g., whether the objectives were clarified); and post-consensus activities. For included articles that assessed the quality of reporting scoping reviews, we planned to abstract the study design, setting, discipline, topic for review, review audience, outcomes (e.g., ability to use review in decision making), and the description of elements used to assess reporting quality (e.g., use of scoping review in title or abstract, protocol mentioned, search strategy, study flow diagram, stakeholder consultation, synthesis methods, and meaning of findings).

The data abstraction form was piloted on a random sample of 10 included articles, and modified as required based on feedback from the team. Full data abstraction began only after sufficient agreement had been obtained (i.e., percent agreement >90 %), which occurred after two rounds of pilot-testing. Subsequently, each included study was abstracted by one team member, and verified by a second reviewer (CN, CW, EL, JPS, KW, MK, RW, WZ). As an additional data cleaning step, a third reviewer (EL, WZ) then verified all changes made by the second reviewer, to ensure data accuracy.

### Methodological quality appraisal

We did not appraise methodological quality or risk of bias of the included articles, which is consistent with guidance on scoping review conduct [3].

### Synthesis

The synthesis included quantitative analysis (e.g., frequency analysis) of the scoping review conduct (i.e., methodological steps) and qualitative analysis (i.e., content analysis) of the components of the research purpose, and conceptual definition of scoping reviews. For the conceptual definition analysis, the definition of a scoping review provided by the author was compared with the research purpose of the scoping review reported in the paper. The items were analysed independently and were subsequently compared by one author (EL), on the basis of how many components matched across the two items. As well, the knowledge translation strategies were classified as integrated knowledge translation and end-of-grant knowledge translation activities. An integrated knowledge translation approach [19, 20] was defined as a collaborative research process whereby researchers and knowledge users work together to design the review, from developing the question through to designing and completing the literature search, analyzing and interpreting the data and disseminating the results. End-of-grant knowledge translation activities [19, 20] were defined as the typical dissemination and implementation activities undertaken by researchers to help ensure that end users are aware of the study findings, beyond publication.

For the qualitative analysis, two authors (WZ, EL) conducted the initial categorization of the key components independently using NVivo 10 [21] and the results were discussed by the team [22]. The team members identified, coded, and charted relevant units of text from the articles using a framework established *a priori* as a guide. The framework was developed through team discussions upon reviewing the preliminary results. Word clouds were drawn using the online program Wordle [23] for the name of the synthesis (by study authors), methodology cited, and frequently cited grey literature sources. This picture displays the frequency of terms, with larger words depicting higher frequency of occurrence. We also conducted a *post hoc* analysis to compare the agreement between methods suggested in the recently published Joanna Briggs Institute guidance [3] and the conduct reported in the included scoping reviews.

## Results

### Literature search

**Fig. 1:**
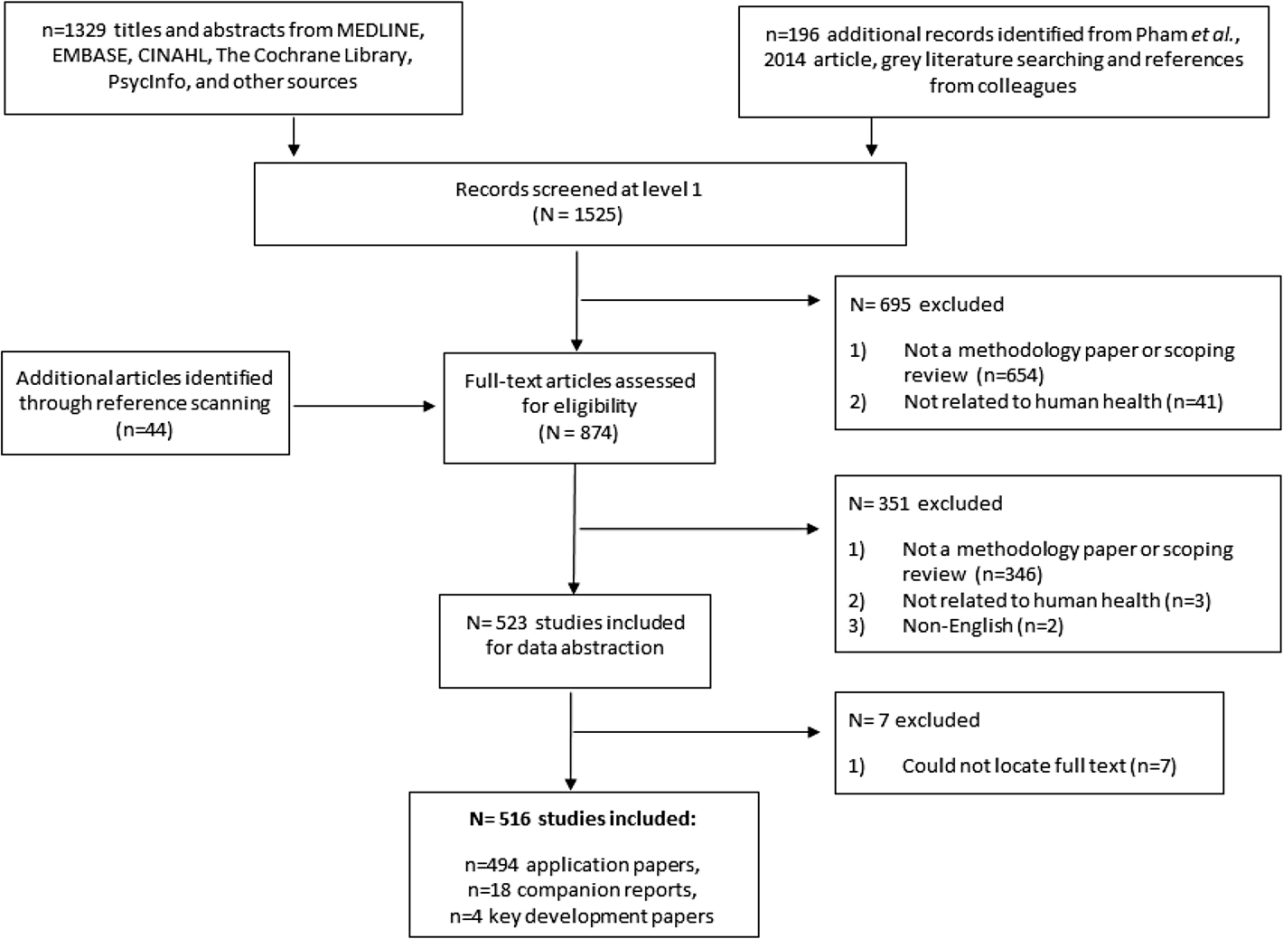
Study flow. Details the flow of information through the different phases of the review; maps out the number of records identified, included and excluded, and the reasons for their exclusion

The literature search resulted in 1525 citations (Fig. 1). After screening 874 potentially relevant full-text papers, 346 were excluded for not being a methodology paper or scoping review, 3 were excluded for not being related to human health, and 2 were excluded for not being written in English. Subsequently, 516 papers were included (full citations listed in Additional file 1: Appendix B, complete data from the included studies are available in Additional file 2). Of these, 4 were papers [1, 2, 10, 24] that described the development of scoping review methodology, 494 were scoping reviews, and 18 were companion reports. All of the 18 companion reports were for the 494 scoping reviews. Approximately 15 % (79/516) were unpublished reports (i.e., grey literature).

### Development papers of scoping review methodology

The four development papers identified were as follows. The Arksey and O’Malley (2005) article was the seminal paper published in 2005, which outlined a framework for conducting scoping studies based on the authors’ experiences of reviewing the literature on services for care-givers in the area of mental health [1]. An article by Andersen et al. (2008) provided an overview of the United Kingdom’s Service Delivery and Organisation Research Programme’s experience with scoping studies; having commissioned a large number of them, including consideration of the key elements in the method, and their impact and use [24]. An article by Levac et al. (2010) put forth specific recommendations to clarify and enhance the methodology for each stage of the Arksey and O’Malley (2005) framework [2]. Lastly, an article by Daudt et al. (2013) discussed the Arksey and O’Malley (2005) framework, and in particular, the team’s experiences using it, in order to develop the methodology further [10].

### Study characteristics

The 494 scoping reviews were disseminated between 1999 and 2014, with 45 % published after 2012 (Table 1). Most were conducted in North America (53 %) and Europe (38 %). Funding was reported in 66 % of the reviews, with the majority being publicly sponsored (64 %). The average size of the scoping review was a mean of 118 included studies (range 1 to 2600).

**Table 1:** Study characteristics

Study Characteristics (*N* = 516)		Count (%)
Year of Publication		
	1999–2003	16 (3 %)
	2004–2008	51 (10 %)
	2009–2012	220 (43 %)
	2013	127 (25 %)
	2014	102 (20 %)
Continent		
	North America	275 (53 %)
	Europe (including UK)	196 (38 %)
	Australia and New Zealand	30 (6 %)
	Asia	9 (2 %)
	Central and South America	3 (1 %)
	Africa	1 (0 %)
	Multiple continents	2 (0 %)
Funding Sources		
	Publicly sponsored	330 (64 %)
	Industry-sponsored	11 (2 %)
	Non-sponsored	25 (5 %)
	Funding not reported	150 (29 %)
Duration of review		
	<6 months	23 (5 %)
	6–12 months	11 (2 %)
	>12 months	7 (1 %)
	Not reported	453 (92 %)
Review Size		
	# of studies: mean (min to max)	449: 117.7 (1 to 2600)

### Terminology and cited framework

**Fig. 2:**
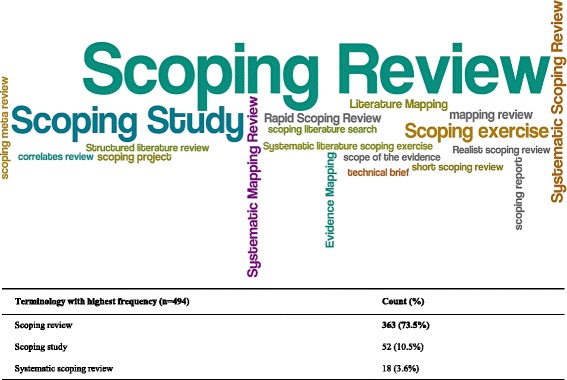
Word cloud of synthesis name. The most commonly used terminology in the 494 scoping reviews is displayed, with the size of the terms in the word cloud corresponding to the frequency of their use

Of the 494 scoping reviews, the most commonly used terminology was “scoping review” (73 %), followed by “scoping study” (10 %; Fig. 2). Arksey and O’Malley (2005) was the most frequently cited framework for guiding the conduct of the scoping review (55 %), followed by Levac et al. (2010) (12 %; Additional file 1: Appendix C).

### Purpose and scoping review definition

Of the 494 scoping reviews, the most common research purpose was to explore the breadth of research (68 %; Additional file 1: Appendix D). For the scoping reviews that provided a definition of a scoping review, the most common component was to map the literature (84 %; Additional file 1: Appendix E). For 12 % of the included scoping reviews, the purpose did not match the conceptual definition of a scoping review, as proposed by study authors (Additional file 1: Appendix F). An example of when the conceptual definition and the research objective(s) did not match is when scoping reviews tended to be described as very similar to systematic reviews, except for the quality appraisal step, whereas the purpose of the study was to explore the breadth of available evidence. This is discrepant given that systematic reviews aim to answer very specific questions and are not exploratory in nature.

### Methodological conduct of the scoping reviews

Thirteen percent (13 %) of the 494 scoping reviews reported having an *a priori* protocol for conducting the scoping review (Table 2). The research question, eligibility criteria, and search strategy were clearly reported in 92 %, 67 % and 22 % of the reviews, respectively. Primary studies were included in 23 % of the reviews. Most authors reported searching more than 1 database (93 %), scanning the reference lists of included studies (56 %), and searching for grey literature (51 %), where grey literature repository and library catalogues (e.g., OpenSigle, Cochrane Library) were the most common types of sources searched (57 %; Additional file 1: Appendix G). Date limitations were employed in 72 % of the scoping reviews, as well as by language in 66 %. In terms of data collection, a predefined abstraction form was mentioned in 43 % of the reviews and quality appraisal was conducted in 14 %.

**Table 2:** Summary of scoping review methods

	Protocol Development & Review Design (*n* = 494)		Count (%)
A priori protocol and review design	Predefined protocol	*A priori* protocol	62 (13 %)
		Not reported	432 (87 %)
	Research Question	Clearly Reported & Iteratively defined	20 (4 %)
		Clearly Reported	456 (92 %)
		Iteratively Defined	2 (<1 %)
		Unclear/inferred	16 (3 %)
	Eligibility Criteria	Clearly Reported & Iteratively defined	54 (11 %)
		Clearly Reported	332 (67 %)
		Iteratively Defined	5 (1 %)
		Unclear/inferred	83 (17 %)
		Not reported	20 (4 %)
	Eligible Study Designs	Primary only (e.g., randomized trials, cohort studies)	113 (23 %)
		Secondary only (e.g., systematic reviews)	14 (3 %)
		Secondary & Primary	82 (17 %)
		All study designs	83 (17 %)
		Not specified	202 (41 %)
Identifying relevant studies	Search Strategy	Clearly Reported & Iteratively defined	43 (9 %)
		Clearly Reported	111 (22 %)
		Keywords only	293 (59 %)
		Iteratively Defined	14 (3 %)
		Unclear/Not reported	33 (7 %)
	Databases searched	Searched >1 database	458 (93 %)
		Searched only 1 database	28 (6 %)
		Searched a selection of journals	3 (1 %)
		Used previous review(s) as starting point	1 (0 %)
		Not reported	4 (1 %)
	Additional search strategy	Scanned references	278 (56 %)
		Grey literature searched	255 (52 %)
		Consulted topic experts	184 (37 %)
		Consulted librarian	135 (27 %)
		Manually searched select Journals	116 (23 %)
		Performed updated search	45 (9 %)
	Limits applied	Limited by date	355 (72 %)
		Limited by language	324 (66 %)
		Limited by study design	54 (11 %)
Data abstraction and Quality appraisal	Standardized charting form	Used a predefined form	212 (43 %)
		Didn’t use predefined form	31 (6 %)
		Not reported	251 (51 %)
	Quality appraisal	Used quality appraisal tool	71(14 %)
		Not done	423 (86 %)
Reporting and Implications of findings	Synthesis	Meta-analysis (i.e. statistical pooling of evidence)	7 (1%)
		Formal qualitative analysis	104 (21 %)
	Reporting	Study flow diagram	232 (47 %)
		Tabular format	403 (82 %)
		Graphical format	83 (17 %)
	Discussion	Identified evidence gaps	420 (85 %)
		Future research opportunity	413 (84 %)
		Strengths and Limitations identified	339 (69 %)
		Specific policy or practice implications	269 (54 %)
		Recommended a systematic review	59 (12 %)
	Knowledge Translation	Integrated and End-of-grant	15 (3 %)
		Integrated	28 (6 %)
		End-of-grant	46 (9 %)

Less than half of the 494 included scoping reviews used a study flow figure (47 %; Table 2). The scoping reviews identified evidence gaps (85 %), future research opportunities (84 %), strengths and limitations (69 %), and implications for policy or practice (54 %). Twelve percent (12 %) recommended a future systematic review. A meta-analysis was conducted in 1 % , while a qualitative analysis (e.g., thematic analysis) was conducted in 21 %.

### Scoping review conduct of published reviews compared with the Joanna Briggs Institute Guidance

Many of the steps recommended by the Joanna Briggs Institute guidance on scoping reviews were not reported by the authors of the 494 included scoping reviews, including: using a protocol (missing in 87 %), having two reviewers independently screen titles/abstracts (missing in 64 %) and screen full-text articles (missing in 71 %), using a predefined charting form (missing in 57 %), and presenting the study flow diagram (missing in 53 %; Table 3, Additional file 1: Appendix H).

**Table 3:** Review process

Review Process (*N* = 494)	Title & Abstract Screening Count (%)	Full-text Screening Count (%)	Data Charting Count (%)
≥2 independent reviewers	167 (34 %)	133 (27 %)	108 (22 %)
1 reviewer & 1 verifier	10 (2 %)	11 (2 %)	43 (9 %)
1 reviewer only	49 (10 %)	32 (6 %)	44 (9 %)
Done but unclear # of reviewers	150 (30 %)	131 (27 %)	186 (38 %)
Not done	2 (0 %)	12 (2 %)	3 (1 %)
Not reported	116 (23 %)	175 (35 %)	110 (22 %)

### Knowledge translation activities

An integrated knowledge translation approach was reported in 6 % of the 494 included scoping reviews (Table 2, Additional file 1: Appendix I). In contrast, end-of-grant knowledge translation activities were reported in 9 % of the reviews. Six percent of the scoping reviews reported using both integrated and end-of-grant knowledge translation strategies. The target audience for the included scoping review was mostly researchers (89 %), healthcare professionals (84 %), government authorities and policy-makers (53 %), and patients (27 %; Table 4).

**Table 4:** Target audience(s)

Most Frequently Reported Target Audiences (*n* = 494)	Count (%)
Researchers (including technology and information specialists)	438 (89 %)
Healthcare and Allied Care Professionals (including managers, program planners, administrators)	415 (84 %)
Government authorities and policy-makers	262 (53 %)
Public Health Professionals (e.g., Epidemiologist, Health Promotion Specialists)	33 (7 %)
Patients and Community Members	27 (5 %)
Educators	25 (5 %)
Social and Community Outreach Worker	22 (4 %)
Funding bodies	11 (2 %)

### Reporting guidance and quality of reporting

We did not identify any guidelines for reporting scoping reviews or studies that assessed the quality of scoping review reporting.

## Discussion

We conducted a comprehensive scoping review that included 4 development papers [1, 2, 10, 24] on scoping reviews and 494 scoping reviews. Our results highlight an explosion in the number of scoping reviews produced since 2012. However, variability in the reporting and conduct of scoping reviews was observed, which may impact health decision-making. Most of the scoping reviews were completed with funding, which was often from a public organization, which suggests that decision-makers are requesting these reviews. As such, improved quality of reporting is imperative for scoping reviews.

As well, our results suggest that the methodology used by the scoping reviews can be improved. When we compared the methods employed by the 494 scoping reviews, we identified a lack of compliance on key items recommended by the Joanna Briggs Institute in their methods guidance for scoping reviews. Indeed, many of the scoping reviews reported shortcuts in their methods, making them similar to those included within our recent scoping review of rapid review methods [25]. However, given that the Joanna Briggs Institute only recently published their methods guidance, this could suggest a lack of awareness of the methodological rigour required to conduct a scoping review, such as the use of a protocol, which was not mentioned in the previous guidance [1]. Taking the newly available guidance into account, a future update of our scoping review will help to identify any improvements in the conduct of scoping reviews.

We are aware of a previous scoping review of scoping reviews [16]. Although this scoping review was not exclusively focused on human health, variable reporting was also observed. Elements that we incorporated in our scoping review that were not found in the previous review by Pham and colleagues include the conduct analysis using the Joanna Briggs Manual, the knowledge translation initiatives analysis, and the comparison of the scoping review conceptual definitions with the scoping review objectives.

The lack of compliance with key steps outlined in the Joanna Briggs Institute manual could also be an issue of poor reporting; specifically, perhaps authors of scoping reviews were not aware of the items that are necessary to report. This is particularly problematic, as 54 % of the included scoping reviews reported some policy implications with respect to their findings. We suggest that further education is necessary for researchers conducting scoping reviews, journal editors, peer reviewers, and funding agencies on the important components of a scoping review. For example, online modules can be shared with these important stakeholders. Since a reporting guideline for scoping reviews was not identified, this is another initiative that may boost reporting of scoping reviews. Members of our research team are currently seeking funding to produce a reporting guideline for scoping reviews.

We interpreted the final step in the Arksey and O’Malley (2005) framework [1], which they call the consultation exercise, as a knowledge translation activity. Surprisingly, very few of the included scoping reviews reported on their consultation exercise or knowledge translation activities. The small proportion of studies with knowledge translation activities could be related to the fact that this step was described as optional in the Arksey and O’Malley (2005) framework [1], or perhaps because authors did not feel it was necessary to report the details concerning this step in their scoping review publications. This step is particularly important if the scoping review was being done for a knowledge user rather than the research team. Occasionally, details about the consultation stage are provided in the discussion section of the manuscript, to provide context for and/or clarify themes apparent in the scoping review findings. Sometimes, the consultation stage may have been done, but published in a subsequent manuscript and not labeled as a scoping review. As such, it might not have been captured in our review.

The consultation exercise has proven to be useful to members of our research team when we have conducted previous scoping reviews [25]. Specific to this scoping review, we conducted a consultation exercise to ensure our results were relevant and to establish our future research agenda. The “Advancing the Field of Scoping Study Methodology” meeting was held on June 8 and 9, 2015 in Toronto, Ontario, Canada. Over 48 participants from Canada, UK, and USA were involved with the scoping review meeting, including researchers, clinicians, students, community organization representatives, people living with chronic disease, and policy makers. A presentation was conducted on our scoping review findings and the participants helped put our findings into context. A separate paper on the results of our consultation exercise has been submitted for publication (O’Brien KK, Colquhoun H, Levac D, Baxter L, Tricco AC, Straus S, et al. (2016). Advancing Scoping Study Methodology: A web-based survey and consultation of perceptions on terminology, definition and methodological steps).

Very few of the scoping reviews (12 %) recommended the conduct of a future systematic review. All of the other included scoping reviews did not comment on the conduct of a systematic review. This may imply that scoping review authors are not using their scoping review to recommend the conduct of a future systematic review. The most common purposes for carrying out a scoping review were to identify evidence gaps and future research opportunities. We found that scoping reviews have been useful for identifying a need for future systematic reviews (e.g., when at least 10 studies are available on a specific topic) and additional study is warranted to examine this association more closely.

Some limitations to our scoping review exist that are worth noting. First, scoping reviews have inherent limitations because the focus is to provide breadth rather than depth of information in a particular topic. As such, the conduct of a meta-analysis is generally not conducted in a scoping review. However, this method was appropriate, given that our objective was to map out the evidence on scoping reviews in the literature. As well, we limited the included studies to those disseminated in English, due to the vast number of included studies. As such, our results are generalizable to scoping reviews written in English.

We anticipate that our results will be of interest to knowledge users, including journal editors, funders, the EQUATOR Network, and researchers who conduct scoping reviews. We plan to use our results to create an online educational module for trainees, peer reviewers, and journal editors on the conduct and reporting of scoping reviews. Our ultimate goal is to create a guideline in the form of a checklist for reporting scoping reviews and their protocols using the methods outlined by the EQUATOR Network [26]. We plan to have the scoping review reporting guideline (and checklist) specific to health research and hosted on the EQUATOR website.

## Conclusions

The number of scoping reviews conducted per year is increasing steadily in recent years. Scoping reviews are used to inform research agendas and identify implications for policy or practice. As such, improvements in the reporting and conduct are imperative. Further research on scoping review methodology is warranted, and in particular, there is need for a guideline to standardize reporting.

## Additional files

Additional file 1: Appendix A. – MEDLINE search strategy: contains the complete search strategy for the MEDLINE database; designed and carried out by an information specialist. **Appendix B** - References to Included Studies. **Appendix C** - Word cloud of methodology cited. **Appendix D** - Components listed in the research objectives: provides a thematic summary of research objectives reported by authors. **Appendix E** - Components listed in the conceptual definitions: contains the main components listed in the conceptual or working definitions of a “scoping review” from the included scoping reviews, along with frequency (i.e. count/proportion) information. **Appendix F** - Agreement between the definition of a scoping review and the research objective(s): provides an overall comparison between research objectives reported and the definition of a scoping review based on the thematic analysis. **Appendix G** - Types of sources searched for grey literature. **Appendix H** - Joanna Briggs Institute Methodology Assessment: An assessment of the methodology of the 494 included scoping reviews relative to each of the steps recommended by the Joanna Briggs Institute guidance on scoping reviews. **Appendix I** - Description of key components of knowledge translation (KT) activities: contains a brief description of the main components of the knowledge translation activities from the included scoping reviews, by category of KT (i.e. integrated KT, end of grant KT, integrated and end of grant KT). (DOCX 802 kb) Additional file 2:
**Data for all included studies in the scoping review of scoping reviews.** This includes all of the data that were abstracted and verified from the included studies. (XLSX 1.26 MB)

## Abbreviations

CINAHL: Cumulative Index to Nursing and Allied Health Literature
EQUATOR: Enhancing the QUAlity and Transparency Of health Research
ERIC: Education Resources Information Center
LISA: Library and Information Science Abstracts
WHO: World Health Organization
