# Supplementary material for: A scoping review on the conduct and reporting of scoping reviews
Source: BMC Med Res Methodol. 2016 Feb 9;16:15. doi: 10.1186/s12874-016-0116-4 (PMC4746911; doi:10.1186/s12874-016-0116-4)
Supplement: Additional file 1: Appendix A. — – MEDLINE search strategy: contains the complete search strategy for the MEDLINE database; designed and carried out by an information specialist. Appendix B - References to Included Studies. Appendix C - Word cloud of methodology cited. Appendix D - Components listed in the research objectives: provides a thematic summary of research objectives reported by authors. Appendix E - Components listed in the conceptual definitions: contains the main components listed in the conceptual or working definitions of a “scoping review” from the included scoping reviews, along with frequency (i.e. count/proportion) information. Appendix F - Agreement between the definition of a scoping review and the research objective(s): provides an overall comparison between research objectives reported and the definition of a scoping review based on the thematic analysis. Appendix G - Types of sources searched for grey literature. Appendix H - Joanna Briggs Institute Methodology Assessment: An assessment of the methodology of the 494 included scoping reviews relative to each of the steps recommended by the Joanna Briggs Institute guidance on scoping reviews. Appendix I - Description of key components of knowledge translation (KT) activities: contains a brief description of the main components of the knowledge translation activities from the included scoping reviews, by category of KT (i.e. integrated KT, end of grant KT, integrated and end of grant KT). (DOCX 802 kb) [file 12874_2016_116_MOESM1_ESM.docx]

Additional File 1: Appendices A-I

[Appendix A. Search Strategy 2](#_Toc439750711)

[Appendix B. References to Included Studies 3](#_Toc439750712)

[Included Studies (n = 516) 3](#_Toc439750713)

[References 3](#_Toc439750714)

[Appendix C. Word cloud of methodology cited 26](#_Toc439750715)

[Appendix D. Purposes of conducting the scoping review 27](#_Toc439750716)

[Appendix E. Elements in the definition of a scoping review provided in the scoping review 28](#_Toc439750717)

[Appendix F. Agreement between the definition of a scoping review and the research objective(s) 29](#_Toc439750718)

[Appendix G. Types of sources searched for grey literature 30](#_Toc439750719)

[Appendix H. Joanna Briggs Institute Methodology Assessment 31](#_Toc439750720)

[Appendix I. Description of key components of knowledge translation activities 32](#_Toc439750721)

# Appendix A. Search Strategy

**2014 Aug 26**

Database: Embase Classic+Embase <1947 to 2014 August 25>, Ovid MEDLINE(R) In-Process & Other Non-Indexed Citations and Ovid MEDLINE(R) <1946 to Present>, PsycINFO <1806 to August Week 3 2014> Search Strategy:

--------------------------------------------------------------------------------

1 (scoping adj2 review).tw.

2 (scoping adj2 reviews).tw.

3 (scoping adj2 overview*).tw.

4 (scoping adj2 search*).tw.

5 (scoping adj2 study).tw.

6 (scoping adj2 studies).tw.

7 (scoping adj2 exercise*).tw.

8 (mapping adj2 review).tw.

9 (mapping adj2 reviews).tw.

10 (mapped adj2 review).tw.

11 (mapped adj2 reviews).tw.

12 (mapping adj2 overview*).tw.

13 (mapped adj2 overview*).tw.

14 (literature adj2 mapping).tw.

15 (correlates adj2 review).tw.

16 (correlates adj2 reviews).tw.

17 or/1-16

18 remove duplicates from 17

# Appendix B. References to Included Studies

## Included Studies (n = 516)

The literature search resulted in 1,551 citations. After screening 900 potentially relevant full-text papers, 516 papers were included [[1-249](#_ENREF_1)][[250-498](#_ENREF_250)][[499-516](#_ENREF_499)].

Of these, 4 were papers [[16](#_ENREF_16), [26](#_ENREF_26), [121](#_ENREF_121), [286](#_ENREF_286)] that described the development of scoping review methodology, 494 were scoping reviews, and 18 were companion reports [[42](#_ENREF_42), [82](#_ENREF_82), [96](#_ENREF_96), [136](#_ENREF_136), [202](#_ENREF_202), [204](#_ENREF_204), [217](#_ENREF_217), [228](#_ENREF_228), [302](#_ENREF_302), [395](#_ENREF_395), [414-419](#_ENREF_414), [421](#_ENREF_421), [422](#_ENREF_422)]. Of the 516 included studies, 79 were identified as unpublished reports (i.e., grey literature) [[10](#_ENREF_10), [25](#_ENREF_25), [27](#_ENREF_27), [32](#_ENREF_32), [35](#_ENREF_35), [41](#_ENREF_41), [57](#_ENREF_57), [60](#_ENREF_60), [79](#_ENREF_79), [80](#_ENREF_80), [95](#_ENREF_95), [96](#_ENREF_96), [99](#_ENREF_99), [102](#_ENREF_102), [106](#_ENREF_106), [113](#_ENREF_113), [124](#_ENREF_124), [136](#_ENREF_136), [143](#_ENREF_143), [144](#_ENREF_144), [149](#_ENREF_149), [155](#_ENREF_155), [160-162](#_ENREF_160), [164](#_ENREF_164), [177](#_ENREF_177), [191](#_ENREF_191), [197](#_ENREF_197), [200](#_ENREF_200), [201](#_ENREF_201), [207](#_ENREF_207), [209](#_ENREF_209), [210](#_ENREF_210), [217](#_ENREF_217), [219](#_ENREF_219), [220](#_ENREF_220), [224](#_ENREF_224), [244](#_ENREF_244), [254](#_ENREF_254), [255](#_ENREF_255), [260](#_ENREF_260), [274](#_ENREF_274), [294](#_ENREF_294), [305](#_ENREF_305), [310](#_ENREF_310), [317](#_ENREF_317), [335](#_ENREF_335), [349](#_ENREF_349), [351](#_ENREF_351), [355](#_ENREF_355), [370](#_ENREF_370), [383](#_ENREF_383), [393](#_ENREF_393), [396](#_ENREF_396), [400](#_ENREF_400), [414-420](#_ENREF_414), [422](#_ENREF_422), [423](#_ENREF_423), [437](#_ENREF_437), [455](#_ENREF_455), [464](#_ENREF_464), [467](#_ENREF_467), [472](#_ENREF_472), [477](#_ENREF_477), [487](#_ENREF_487), [492](#_ENREF_492), [495](#_ENREF_495), [499](#_ENREF_499), [505](#_ENREF_505), [506](#_ENREF_506), [511](#_ENREF_511), [514](#_ENREF_514)]

## References

1. Aarts J, Van den Haak P, Nelen W, Tuil W, Faber M, Kremer J. Patient-focused internet interventions in reproductive medicine: a scoping review. *Human reproduction update.* 2012; 18(2):211-227.

2. Abbott P, Liu Y. A scoping review of telehealth. *Yearbook of medical informatics.* 2013; 8(1):51-58.

3. Abboud J, Marchand AA, Sorra K, Descarreaux M. Musculoskeletal physical outcome measures in individuals with tension-type headache: a scoping review. *Cephalalgia.* 2013; 33(16):1319-1336.

4. Abraham A, Sommerhalder K, Abel T. Landscape and well-being: a scoping study on the health-promoting impact of outdoor environments. *International Journal of Public Health.* 2010; 55(1):59-69.

5. Adams J, White M. Are the stages of change socioeconomically distributed? A scoping review. *American Journal of Health Promotion.* 2007; 21(4):237-247.

6. Affoo RH, Foley N, Rosenbek J, Shoemaker JK, Martin RE. Swallowing dysfunction and autonomic nervous system dysfunction in Alzheimer's disease: a scoping review of the evidence. *Journal of the American Geriatrics Society.* 2013; 61(12):2203-2213.

7. Afzal Z, Muntaner C, Chung H, Mahmood Q, Ng E, Schrecker T. Complementarities or contradictions? Scoping the health dimensions of "flexicurity" labor market policies. *International Journal of Health Services.* 2013; 43(3):473-482.

8. Agomo CO. The role of community pharmacists in public health: a scoping review of the literature. *Journal of Pharmaceutical Health Services Research.* 2012; 3(1):25-33.

9. Ahmed T, Lucas H, Khan AS, Islam R, Bhuiya A, Iqbal M. eHealth and mHealth initiatives in Bangladesh: A scoping study. *BMC health services research.* 2014; 14(1):260.

10. Akpokodje J, Bowles R, Tigere E. Evidence-based approaches to crime prevention in developing countries-A scoping review of the literature. In*.*: Centre for Criminal Justice Economics and Psychology; 2002.

11. Alam R, Speed S, Beaver K. A scoping review on the experiences and preferences in accessing diabetes-related healthcare information and services by British Bangladeshis. *Health and Social Care in the Community.* 2012; 20(2):155-171.

12. Algilani S, Östlund-Lagerström L, Kihlgren A, Blomberg K, Brummer RJ, Schoultz I. Exploring the concept of optimal functionality in old age. *Journal of multidisciplinary healthcare.* 2014; 7:69.

13. Allen-Scott L, Hatfield J, McIntyre L. A scoping review of unintended harm associated with public health interventions: towards a typology and an understanding of underlying factors. *International Journal of Public Health.* 2014; 59(1):3-14.

14. Aminzadeh F, Molnar FJ, Dalziel WB, Ayotte D. A review of barriers and enablers to diagnosis and management of persons with dementia in primary care. *Canadian Geriatrics Journal.* 2012; 15(3):85-94.

15. Anaby D, Hand C, Bradley L, DiRezze B, Forhan M, DiGiacomo A *et al*. The effect of the environment on participation of children and youth with disabilities: a scoping review. *Disability and Rehabilitation.* 2013; 35(19):1589-1598.

16. Anderson S, Allen P, Peckham S, Goodwin N. Asking the right questions: scoping studies in the commissioning of research on the organisation and delivery of health services. *Health Research Policy and Systems.* 2008; 6(7):1-12.

17. Anema A, Marshall BD, Stevenson B, Gurm J, Montaner G, Small W *et al*. Intergenerational sex as a risk factor for HIV among Young Men Who Have Sex with Men: A scoping review. *Current HIV/AIDS Reports.* 2013; 10(4):398-407.

18. Anstee S, Price A, Young A, Barnard K, Coates B, Fraser S *et al*. Developing a matrix to identify and prioritise research recommendations in HIV prevention. *BMC Public Health.* 2011; 11:1-8.

19. Antao L, Shaw L, Ollson K, Reen K, To F, Bossers A *et al*. Chronic pain in episodic illness and its influence on work occupations: a scoping review. *Work.* 2013; 44(1):11-36.

20. Arai L, Stapley S, Roberts H. 'Did not attends' in children 0-10: a scoping review. *health and development.* 2014; 40(6):797-805.

21. Archambault C, Arel R, Filion KB. Gestational diabetes and risk of cardiovascular disease: a scoping review. *Open Medicine.* 2014; 8(1):e1-e9.

22. Archambault PM, Van de Belt TH, Grajales FJI, Faber MJ, Kuziemsky CE, Gagnon S *et al*. Wikis and collaborative writing applications in health care: A scoping review. *Journal of Medical Internet Research.* 2013; 15(10):4-38.

23. Archer N, Fevrier-Thomas U, Lokker C, McKibbon KA, Straus SE. Personal health records: a scoping review. *Journal of the American Medical Informatics Association.* 2011; 18(4):515-522.

24. Arksey H. Scoping the field: services for carers of people with mental health problems. *Health & social care in the community.* 2003; 11(4):335-344.

25. Arksey H, Corden A, Glendinning C, Hirst M. Minding the Money: Carers and the Management of Financial Assets in Later Life. In*.*: Social Policy Research Unit, University of York; 2006.

26. Arksey H, O'Malley L. Scoping studies: Towards a Methodological Framework. *International journal of social research methodology.* 2005; 8(1):19-32.

27. Arksey H, O'Malley L, Baldwin S, Harris J, Mason A, Golder S. Literature Review Report: Services to support carers of people with mental health problems. In*.* London; 2002.

28. Armstrong R, Waters E, Dobbins M, Anderson L, Moore L, Petticrew M *et al*. Knowledge translation strategies to improve the use of evidence in public health decision making in local government: intervention design and implementation plan. *Implementation Science.* 2013; 8(1):121.

29. Asano M, Berg E, Johnson K, Turpin M, Finlayson ML. A scoping review of rehabilitation interventions that reduce fatigue among adults with multiple sclerosis. *Disabil Rehabil.* 2015; 37(9):729-738.

30. Asano M, Raszewski R, Finlayson M. Rehabilitation interventions for the management of multiple sclerosis relapse: a short scoping review. *International Journal of MS care.* 2014; 16(2):99-104.

31. Ashcroft R, Silveira J, Rush B, McKenzie K. Incentives and disincentives for the treatment of depression and anxiety: a scoping review. *Canadian Journal of Psychiatry.* 2014; 59(7):385-392.

32. Atkinson M. Safeguarding post-Laming: initial scoping study (Local Government Association Research Report). In*.* Slough; 2010: 1-25.

33. Babinec PM, Rock MJ, Lorenzetti DL, Johnson JA. Do researchers use pharmacists' communication as an outcome measure? A scoping review of pharmacist involvement in diabetes care. *International Journal of Pharmacy Practice.* 2010; 18(4):183-193.

34. Bagaria J, Heggie C, Abrahams J, Murray V. Evacuation and sheltering of hospitals in emergencies: a review of international experience. *Prehospital and Disaster Medicine.* 2009; 24(5):461-467.

35. Bagnall A, Wilby J, Glanville J, Sowden A. Scoping review of sabotage and/or tampering in the NHS. York, UK: University of York; 2004.

36. Bamm EL, Rosenbaum P, Wilkins S. Is Health Related Quality Of Life of people living with chronic conditions related to patient satisfaction with care? *Disability and Rehabilitation.* 2013; 35(9):766-774.

37. Banner D, MacLeod ML, Johnston S. Role transition in rural and remote primary health care nursing: a scoping literature review. *Canadian Journal of Nursing Research.* 2010; 42(4):40-57.

38. Barbic SP, Bartlett SJ, Mayo NE. Emotional vitality: concept of importance for rehabilitation. *Archives of Physical Medicine and Rehabilitation.* 2013; 94(8):1547-1554.

39. Bassi J, Lau F. Measuring value for money: a scoping review on economic evaluation of health information systems. *Journal of the American Medical Informatics Association.* 2013; 20(4):792-801.

40. Bassi J, Lau F, Bardal S. Use of information technology in medication reconciliation: a scoping review. *Annals of Pharmacotherapy.* 2010; 44(5):885-897.

41. Baxter K, Glendinning C, Clarke S. Scoping Review on Access to Information about Social Care Services. In*.*: Social Policy Research Unit, University of York; 2006.

42. Baxter K, Glendinning C, Clarke S. Making informed choices in social care: the importance of accessible information. *Health Soc Care Community.* 2008; 16(2):197-207.

43. Bell CA, Duncan G, Saini B. Knowledge, attitudes and practices of private sector providers of tuberculosis care: a scoping review. *International Journal of Tuberculosis and Lung Disease.* 2011; 15(8):1005-1017.

44. Bender A, Guruge S, Hyman I, Janjua M. Tuberculosis and common mental disorders: international lessons for Canadian immigrant health. *Canadian Journal of Nursing Research.* 2012; 44(4):56-75.

45. Bennett KM, Scornaiencki JM, Brzozowski J, Denis S, Magalhaes L. Immigration and its impact on daily occupations: A scoping review. *Occupational therapy international.* 2012; 19(4):185-203.

46. Bernardin KN, Toews DN, Restall GJ, Vuongphan L. Self-management interventions for people living with human immunodeficiency virus: a scoping review. *Canadian Journal of Occupational Therapy.* 2013; 80(5):314-327.

47. Bernardo TM, Rajic A, Young I, Robiadek K, Pham MT, Funk JA. Scoping review on search queries and social media for disease surveillance: a chronology of innovation. *Journal of Medical Internet Research.* 2013; 15(7):e147, 2013.

48. Bertelli M, Merli MP, Rossi M, Lassi S, Bianco A, Colangelo J. Quality of life in pharmacological intervention on autism spectrum disorders. *Advances in Mental Health and Intellectual Disabilities.* 2013; 7(1):40-48.

49. Birnbaum R, Saini M. A scoping review of qualitative studies about children experiencing parental separation. *Childhood.* 2012:0907568212454148.

50. Bish M, Kenny A, Nay R. A scoping review identifying contemporary issues in rural nursing leadership. *Journal of Nursing Scholarship.* 2012; 44(4):411-417.

51. Bisht R, Pitchforth E, Murray SF. Understanding India, globalisation and health care systems: a mapping of research in the social sciences. *Globalization and Health.* 2012; 8(1):1-15.

52. Boeltzig H, Pilling D, Timmons JC, Johnson R. Disability Specialist Staff in US One-Stop Career Centers and British Jobcentre Plus Offices: Roles, Responsibilities, and Evidence of Their Effectiveness. *Journal of Disability Policy Studies.* 2010; 21(2):101-115.

53. Bogo M, Shlonsky A, Lee B, Serbinski S. Acting Like It Matters: A Scoping Review of Simulation in Child Welfare Training. *Journal of Public Child Welfare.* 2014; 8(1):70-93.

54. Borkhoff CM, Wieland ML, Myasoedova E, Ahmad Z, Welch V, Hawker GA *et al*. Reaching those most in need: a scoping review of interventions to improve health care quality for disadvantaged populations with osteoarthritis. *Arthritis care & research.* 2011; 63(1):39-52.

55. Borschmann R, Greenberg N, Jones N, Henderson RC. Campaigns to reduce mental illness stigma in Europe: A scoping review. *Die Psychiatrie: Grundlagen and Perspektiven.* 2014; 11(1):43-50.

56. Borthwick R, Newbronner L, Stuttard L. ‘Out of Hospital’: a scoping study of services for carers of people being discharged from hospital. *Health & social care in the community.* 2009; 17(4):335-349.

57. Bostock L, Brodie I, Clapton J, Fish S, Fisher M, Morris M *et al*. Increasing the number of careleavers in ‘settled, safe accommodation’ (C4EO Scoping Review 3). In*.*; 2009.

58. Bostrom AM, Slaughter SE, Chojecki D, Estabrooks CA. What do we know about knowledge translation in the care of older adults? A scoping review. *Journal of the American Medical Directors Association.* 2012; 13(3):210-219.

59. Bottorff JL, Oliffe JL, Robinson CA, Carey J. Gender relations and health research: a review of current practices. *International Journal for Equity in Health.* 2011; 10(1):60.

60. Boyd A, Chambers N, French S, King R, Shaw D, Whitehead M. A scoping study of emergency planning and management in health care: What further research is needed? In*.*; 2012.

61. Boydell KM, Gladstone BM, Volpe T, Allemang B, Stasiulis E. The Production and Dissemination of Knowledge: A Scoping Review of Arts-Based Health Research. *2012.* 2012; 13(1).

62. Bragge P, Clavisi O, Turner T, Tavender E, Collie A, Gruen RL. The Global Evidence Mapping Initiative: scoping research in broad topic areas. *BMC Med Res Methodol.* 2011; 11:1-12.

63. Branas P, Jordan R, Fry-Smith A, Burls A, Hyde C. Treatments for fatigue in multiple sclerosis: a rapid and systematic review. *Health Technology Assessment.* 2000; 4(27):1-61.

64. Brandt B, Lutfiyya MN, King JA, Chioreso C. A scoping review of interprofessional collaborative practice and education using the lens of the Triple Aim. *Journal of Interprofessional Care.* 2014; 28(5):393-399.

65. Brennan N, Barnes R, Calnan M, Corrigan O, Dieppe P, Entwistle V. Trust in the health-care provider-patient relationship: a systematic mapping review of the evidence base. *International Journal for Quality in Health Care.* 2013; 25(6):682-688.

66. Brennan R, Van Hout MC. Gamma-Hydroxybutyrate (GHB): A Scoping Review of Pharmacology, Toxicology, Motives for Use, and User Groups. *Journal of Psychoactive Drugs.* 2014; 46(3):243-251.

67. Brien S, Gheihman G, Tse YK, Byrnes M, Harrison S, Dobrow MJ. A scoping review of appropriateness of care research activity in Canada from a health system-level perspective. *Healthcare Policy.* 2014; 9(4):48-61.

68. Brien SE, Lorenzetti DL, Lewis S, Kennedy J, Ghali WA. Overview of a formal scoping review on health system report cards. *Implementation Science.* 2010; 5(2):2.

69. Brouwers MC, De Vito C, Bahirathan L, Carol A, Carroll JC, Cotterchio M *et al*. What implementation interventions increase cancer screening rates? A systematic review. *Implementation Science.* 2011; 6(1):111.

70. Broyles LM, Conley JW, Harding JD, Jr., Gordon AJ. A scoping review of interdisciplinary collaboration in addictions education and training. *Journal of Addictions Nursing.* 2013; 24(1):29-36.

71. Bryden A, Petticrew M, Mays N, Eastmure E, Knai C. Voluntary agreements between government and business - a scoping review of the literature with specific reference to the Public Health Responsibility Deal. *Health Policy.* 2013; 110(2-3):186-197.

72. Bryson-Campbell M, Shaw L, O'Brien J, Holmes J, Magalhaes L. A scoping review on occupational and self identity after a brain injury. *Work.* 2013; 44(1):57-67.

73. Burns TE, Wade J, Stephen C, Toews L. A scoping analysis of peer-reviewed literature about linkages between aquaculture and determinants of human health. *Ecohealth.* 2014; 11(2):227-240.

74. Büscher T, Dyson J, Cowdell F. The effects of hoarding disorder on families: an integrative review. *Journal of Psychiatric and Mental Health Nursing.* 2014; 21(6):491-498.

75. Byrne A. What factors influence the decisions of parents of children with special educational needs when choosing a secondary educational provision for their child at change of phase from primary to secondary education? A review of the literature. *Journal of Research in Special Educational Needs.* 2013; 13(2):129-141.

76. Cahill J, Barkham M, Hardy G, Gilbody S, Richards D, Bower P *et al*. A review and critical appraisal of measures of therapist-patient interactions in mental health settings. *Health Technol Assess.* 2008; 12(24):iii, ix-47.

77. Callahan P, Liu P, Purcell R, Parker AG, Hetrick SE. Evidence map of prevention and treatment interventions for depression in young people. *Depress Res Treat.* 2012; 2012:820735.

78. Cameron JI, Tsoi C, Marsella A. Optimizing stroke systems of care by enhancing transitions across care environments. *Stroke.* 2008; 39(9):2637-2643.

79. Canadian Population Health Initiative (CPHI). Urban Physical environments and Health Inequalities: A Scoping Review of Interventions. In*.*; 2011.

80. Carr-Hill R, Currie L, Dixon P. Skill Mix in Secondary Care: A scoping exercise. In*.*: Centre for Health Economics, University of York; 2003.

81. Carrier A, Levasseur M, Bedard D, Desrosiers J. Community occupational therapists’ clinical reasoning: identifying tacit knowledge. *Australian occupational therapy journal.* 2010; 57(6):356-365.

82. Carter N, Martin-Misener R, Kilpatrick K, Kaasalainen S, Donald F, Bryant-Lukosius D *et al*. The role of nursing leadership in integrating clinical nurse specialists and nurse practitioners in healthcare delivery in Canada. *Nurs Leadersh (Tor Ont).* 2010; 23:167-185.

83. Casey AF. Measuring body composition in individuals with intellectual disability: a scoping review. *Journal of obesity.* 2013; 2013.

84. Casey AF, Rasmussen R. Reduction measures and percent body fat in individuals with intellectual disabilities: a scoping review. *Disability and Health Journal* 2013; 6(1):2-7.

85. Catts SV, O'Toole BI, Carr VJ, Lewin T, Neil A, Harris MG *et al*. Appraising evidence for intervention effectiveness in early psychosis: conceptual framework and review of evaluation approaches. *Australian and New Zealand Journal of Psychiatry.* 2010; 44(3):195-219.

86. Challen K, Goodacre SW. Predictive scoring in non-trauma emergency patients: a scoping review. *Emergency Medicine Journal.* 2011; 28(10):827-837.

87. Challen K, Lee AC, Booth A, Gardois P, Woods HB, Goodacre SW. Where is the evidence for emergency planning: a scoping review. *BMC public health.* 2012; 12(1):542.

88. Chambers LA, Wilson MG, Rueda S, Gogolishvili D, Shi MQ, Rourke SB. Evidence informing the intersection of HIV, aging and health: a scoping review. *AIDS and behavior.* 2014; 18(4):661-675.

89. Cheung G, Pizzola L, Keller H. Dietary, food service, and mealtime interventions to promote food intake in acute care adult patients. *Journal of nutrition in gerontology and geriatrics.* 2013; 32(3):175-212.

90. Cho DB, Cole D, Simiyu K, Luong W, Neufeld V. Mentoring, training and support to global health innovators: a scoping review. *Global Journal of Health Science.* 2013; 5(5):162-173.

91. Chretien JP, George D, Shaman J, Chitale RA, McKenzie FE. Influenza forecasting in human populations: a scoping review. *PLoS One.* 2014; 9(4):e94130, 92014.

92. Churchill P, Otal D, Pemberton J, Ali A, Flageole H, Walton JM. Sclerotherapy for lymphatic malformations in children: a scoping review. *Journal of Pediatric Surgery.* 2011; 46(5):912-922.

93. Cleaver K. Attitudes of emergency care staff towards young people who self-harm: a scoping review. *International Emergency Nursing.* 2014; 22(1):52-61.

94. Cleaver S, Nixon S. A scoping review of 10 years of published literature on community-based rehabilitation. *Disability and Rehabilitation.* 2014; 36(17):1385-1394.

95. Clift S, Hancox G, Staricoff R, Whitmore C. Singing and Health: A Systematic Mapping and Review of Non-Clinical Research. In*.*: Canterbury Christ Church University: Sidney De Haan Research Centre for Arts and Health; 2008.

96. Clift S, Nicol J, Raisbeck M, Whitmore C, Morrison I. Group singing, wellbeing and health: A systematic mapping of research evidence. In*.*; 2010.

97. Coad JE, Shaw KL. Is children's choice in health care rhetoric or reality? A scoping review. *Journal of Advanced Nursing.* 2008; 64(4):318-327.

98. Coast E, Leone T, Hirose A, Jones E. Poverty and postnatal depression: a systematic mapping of the evidence from low and lower middle income countries. *Health & Place.* 2012; 18(5):1188-1197.

99. Cobban SJ: Improving oral health for elderly residents of long-term care facilities. U Alberta; 2013.

100. Coe JB, Young I, Lambert K, Dysart L, Nogueira BL, Rajic A. A scoping review of published research on the relinquishment of companion animals. *Journal of Applied Animal Welfare Science.* 2014; 17(3):253-273.

101. Cogan AM. Occupational needs and intervention strategies for military personnel with mild traumatic brain injury and persistent post-concussion symptoms: a review. *OTJR: occupation, participation and health.* 2014; 34(3):150-159.

102. Coghlan M, Sanders D, Morris M, O'Donnell L, Benefield P, Harper A *et al*. Narrowing the gap in outcomes for children from the most excluded families through inclusive practice in early years settings (C4EO Scoping Review 3). In*.*; 2008.

103. Colquhoun HL, Letts LJ, Law MC, MacDermid JC, Missiuna CA. A scoping review of the use of theory in studies of knowledge translation. *Canadian Journal of Occupational Therapy.* 2010; 77(5):270-279.

104. Constand MK, MacDermid JC. Applications of the International Classification of Functioning, Disability and Health in goal-setting practices in healthcare. *Disability and Rehabilitation.* 2014; 36(15):1305-1314.

105. Constand MK, MacDermid JC, Dal Bello-Haas V, Law M. Scoping review of patient-centered care approaches in healthcare. *BMC health services research.* 2014; 14(1):271.

106. Cornejo E, Denman CA, Sabo S, de Zapién J, Rosales C. Scoping review of community health worker/promotora-based chronic disease primary prevention programs on the US-Mexico border. In*.*; 2011.

107. Costantino MA, Bonati M. A scoping review of interventions to supplement spoken communication for children with limited speech or language skills. *PLoS One.* 2014; 9(3):e90744, 92014.

108. Courtenay M, Conrad P, Wilkes M, La Ragione R, Fitzpatrick N. Interprofessional initiatives between the human health professions and veterinary medical students: a scoping review. *Journal of interprofessional care.* 2014; 28(4):323-330.

109. Courtenay M, Nancarrow S, Dawson D. Interprofessional teamwork in the trauma setting: a scoping review. *Human Resources for Health* 2013; 11(1):57.

110. Craig DG. Current occupational therapy publications in home health: a scoping review. *American Journal of Occupational Therapy.* 2012; 66(3):338-347.

111. Cramm HA, Krupa TM, Missiuna CA, Lysaght RM, Parker KH. Executive functioning: a scoping review of the occupational therapy literature. *Canadian Journal of Occupational Therapy.* 2013; 80(3):131-140.

112. Crawford F. Clinical trials in dental primary care: what research methods have been used to produce reliable evidence? *British Dental Journal.* 2005; 199(3):155-160.

113. Crilly T, Jashapara A, Ferlie E. Research utilisation & knowledge mobilisation: A scoping review of the literature. In*.*; 2010.

114. Cronin S, Curran J, Iantorno J, Murphy K, Shaw L, Boutcher N *et al*. Work capacity assessment and return to work: a scoping review. *Work.* 2013; 44(1):37-55.

115. Crooks VA, Kingsbury P, Snyder J, Johnston R. What is known about the patient's experience of medical tourism? A scoping review. *BMC Health Services Research.* 2010; 10(1):266.

116. Cross A, Rosenbaum P, Gorter JW. Exploring the aquatic environment for disabled children: How we can conceptualize and advance interventions with the ICF. *Critical Reviews in Physical and Rehabilitation Medicine.* 2013; 25(1-2):59-76.

117. Curran C, Burchardt T, Knapp M, McDaid D, Li B. Challenges in Multidisciplinary Systematic Reviewing: A Study on Social Exclusion and Mental Health Policy. *Social Policy and Administration.* 2007; 41(3):289-312.

118. Czekanski KE, Wolf ZR. Encouraging and Evaluating Class Participation. *Journal of University Teaching and Learning Practice.* 2013; 10(1):7.

119. Dagenais C, Malo M, Robert E, Ouimet M, Berthelette D, Ridde V. Knowledge transfer on complex social interventions in public health: a scoping study. *PLoS One.* 2013; 8(12):e80233, 82013.

120. Dalmer N. Health Literacy Promotion: Contemporary Conceptualizations and Current Implementations in Canadian Health Librarianship. *Journal of the Canadian Health Libraries Association.* 2013; 34(1):12-16.

121. Daudt HM, Van Mossel C, Scott SJ. Enhancing the scoping study methodology: a large, inter-professional team’s experience with Arksey and O’Malley’s framework. *BMC medical research methodology.* 2013; 13(1):48.

122. Davis K, Drey N, Gould D. What are scoping studies? A review of the nursing literature. *International Journal of Nursing Studies.* 2009; 46(10):1386-1400.

123. Davis KJ, Kumar D, Wake MC. Pelvic floor dysfunction: a scoping study exploring current service provision in the UK, interprofessional collaboration and future management priorities. *International Journal of Clinical Practice.* 2010; 64(12):1661-1670.

124. Davis P, Florian L. Teaching Strategies and Approaches for Pupils with Special Educational Needs: A Scoping Study (Research Report No 516). In*.*; 2004.

125. Davison SN, Koncicki H, Brennan F. Pain in chronic kidney disease: a scoping review. *Seminars in Dialysis.* 2014; 27(2):188-204.

126. de Chavez AC, Backett-Milburn K, Parry O, Platt S. Understanding and researching wellbeing: Its usage in different disciplines and potential for health research and health promotion. *Health Education Journal.* 2005; 64(1):70-87.

127. Debono DS, Greenfield D, Travaglia JF, Long JC, Black D, Johnson J *et al*. Nurses’ workarounds in acute healthcare settings: a scoping review. *BMC Health Services Research.* 2013; 13(1):175.

128. Decaria JE, Sharp C, Petrella RJ. Scoping review report: obesity in older adults. *International Journal of Obesity.* 2012; 36(9):1141-1150.

129. Deen J, Matos LC, Temple B, Su JY, da SJ, Liberato S *et al*. Identifying national health research priorities in Timor-Leste through a scoping review of existing health data. *Health Research Policy and Systems.* 2013; 11(1).

130. Dellimore K, Helyer A, Franklin S. A scoping review of important urinary catheter induced complications. *Journal of Materials Science: Materials in Medicine.* 2013; 24(8):1825-1835.

131. Denford S, Frost J, Dieppe P, Cooper C, Britten N. Individualisation of drug treatments for patients with long-term conditions: a review of concepts. *BMJ open.* 2013; 4(3):e004172-e004172.

132. Denning DW, Pleuvry A, Cole DC. Global burden of chronic pulmonary aspergillosis as a sequel to pulmonary tuberculosis. *Bulletin of the World Health Organization.* 2011; 89(12):864-872.

133. Denning DW, Pleuvry A, Cole DC. Global burden of allergic bronchopulmonary aspergillosis with asthma and its complication chronic pulmonary aspergillosis in adults. *Med Mycol.* 2013; 51(4):361-370.

134. Deshpande A, Khoja S, Lorca J, McKibbon A, Rizo C, Husereau D *et al*. Asynchronous telehealth: a scoping review of analytic studies. *Open Medicine.* 2009; 3(2):e69-e91.

135. Deutschlander S, Suter E, Grymonpre R. Interprofessional practice education: is the ‘interprofessional’component relevant to recruiting new graduates to underserved areas. *Rural and remote health.* 2013; 13(2489).

136. DiCenso A, Bryant-Lukosius D. Clinical Nurse Specialists and Nurse Practitioners in Canada: A Decision Support Synthesis. In*.*: Canadian Health Services Research Foundation; 2010.

137. DiCenso A, Martin-Misener R, Bryant-Lukosius D, Bourgeault I, Kilpatrick K, Donald F *et al*. Advanced practice nursing in Canada: overview of a decision support synthesis. *Nurs Leadersh (Tor Ont).* 2010; 23:15-34.

138. Dowrick C, Gask L, Edwards S, Aseem S, Bower P, Burroughs H *et al*. Researching the mental health needs of hard-to-reach groups: managing multiple sources of evidence. *BMC Health Services Research.* 2009; 9(1):226.

139. Drager KD, Reichle J, Pinkoski C. Synthesized speech output and children: a scoping review. *American Journal of Speech-Language Pathology.* 2010; 19(3):259-273.

140. Dryden R, Williams B, McCowan C, Themessl-Huber M. What do we know about who does and does not attend general health checks? Findings from a narrative scoping review. *BMC Public Health.* 2012; 12(1):723.

141. Du Toit R, Faal HB, Etya’ale D, Wiafe B, Mason I, Graham R *et al*. Evidence for integrating eye health into primary health care in Africa: a health systems strengthening approach. *BMC health services research.* 2013; 13(1):102.

142. Duffett M, Choong K, Hartling L, Menon K, Thabane L, Cook DJ. Randomized controlled trials in pediatric critical care: a scoping review. *Critical care.* 2013; 17(5):R256.

143. Dutton R. 'Extra care' housing and people with dementia. What do we know about what works regarding the build and social environment and the provision of care and support? A scoping review of the literature 1998-2008. In*.* London; 2009.

144. Elliott B, Scott, A., Skatun, D., Farrar, S., & Napper, M. . The impact of local labour market factors on the organisation and delivery of health services. In: *NHS Service Delivery and Organisation R&D Program.* London; 2003.

145. Elwy AR, Groessl EJ, Eisen SV, Riley KE, Maiya M, Lee JP *et al*. A systematic scoping review of yoga intervention components and study quality. *American Journal of Preventive Medicine.* 2014; 47(2):220-232.

146. Embi P. Clinical research informatics: survey of recent advances and trends in a maturing field. *Yearbook of medical informatics.* 2013; 8(1):178.

147. Emmerick ICM, Oliveira MA, Luiza VL, Azeredo TB, Bigdeli M. Access to medicines in Latin America and the Caribbean (LAC): a scoping study. *BMJ open.* 2013; 3(5):e002224.

148. Fang ML, Gerbrandt J, Liwander A, Pederson A. Exploring promising gender-sensitive tobacco and alcohol use interventions: results of a scoping review. *Substance Use & Misuse.* 2014; 49(11):1400-1416.

149. Farrar AM: The application of research synthesis methods for evaluating primary research on salmonella in broiler chickens. The University of Guelph; 2009.

150. Fathima M, Naik-Panvelkar P, Saini B, Armour CL. The role of community pharmacists in screening and subsequent management of chronic respiratory diseases: a systematic review. *Pharmacy Practice.* 2013; 11(4):228.

151. Faulkner GE, Grootendorst P, Nguyen VH, Andreyeva T, Arbour-Nicitopoulos K, Auld MC *et al*. Economic instruments for obesity prevention: results of a scoping review and modified Delphi survey. *International Journal of Behavioral Nutrition & Physical Activity.* 2011; 8:109.

152. Fayter D, Corbett M, Heirs M, Fox D, Eastwood A. A systematic review of photodynamic therapy in the treatment of pre-cancerous skin conditions, Barrett's oesophagus and cancers of the biliary tract, brain, head and neck, lung, oesophagus and skin. *Health Technology Assessment.* 2010; 14(37):1-288.

153. Feehan LM, Beck CA, Harris SR, MacIntyre DL, Li LC. Exercise prescription after fragility fracture in older adults: a scoping review. *Osteoporosis International.* 2011; 22(5):1289-1322.

154. Flowerdew L, Brown R, Vincent C, Woloshynowych M. Identifying nontechnical skills associated with safety in the emergency department: a scoping review of the literature. *Annals of Emergency Medicine.* 2012; 59(5):386-394.

155. Forbes A, Hughes J, Ismail K, While A. The organization & delivery of diabetes services in the UK: a scoping exercise. In*.*; 2010.

156. Forbes A, While A, Ullman R, Murgatroyd B. The contribution of nurses to child health and child health services: findings of a scoping exercise. *Journal of Child Health Care.* 2007; 11(3):231-247.

157. Forhan M. Weight Loss Interventions for Rehabilitation Patients with Obesity. *Current Obesity Reports.* 2014; 3(3):330-335.

158. Forhan M, Gill S. Cross-Border Contributions to Obesity Research and Interventions: A Review of Canadian and American Occupational Therapy Contributions. *Occupational therapy in health care.* 2013; 27(2):129-141.

159. Forsman H, Vinnerljung B. Interventions aiming to improve school achievements of children in out-of-home care: A scoping review. *Children and Youth Services Review.* 2012; 34(6):1084-1091.

160. Foster C, Amir Z, Jarrett N, Stamataki Z, Brearley S, Scott I. Scoping of Research Evidence Regarding the Health and Well-Being of Cancer Survivors: Psychological and Social Problems Faced by Cancer Survivors, and their Physical and Practical Well-Being. In*.*; 2009.

161. Fotaki M, Boyd A, Smith L, McDonald R, Roland M, Sheaff R *et al*. Patient choice and the organisation and delivery of health services: scoping review. In*.*; 2005.

162. Foundation for People with Learning Disabilities. Equalities Scoping Study. In*.*; 2010: 16.

163. Frasca D, Tomaszczyk J, McFadyen BJ, Green RE. Traumatic brain injury and post-acute decline: what role does environmental enrichment play? A scoping review. *Frontiers in human neuroscience.* 2013; 7.

164. Freeman G, Shepperd S, I R, K E, Richards S. Continuity of Care: Report of a Scoping Exercise. In*.*: National Co-ordinating Centre for NHS Service Delivery and Organisation R & D (NCCSDO); 2001.

165. Fritz CE, Schuurman N, Robertson C, Lear S. A scoping review of spatial cluster analysis techniques for point-event data. *Geospatial Health.* 2013; 7(2):183-198.

166. Frost R, MacPherson H, O'Meara S. A critical scoping review of external uses of comfrey (Symphytum spp.). *Complementary Therapies in Medicine.* 2013; 21(6):724-745.

167. Futamura M, Thomas KS, Grindlay DJ, Doney EJ, Torley D, Williams HC. Mapping systematic reviews on atopic eczema-an essential resource for dermatology professionals and researchers. *PLoS One.* 2013; 8(3):e58484.

168. Gagliardi AR, Brouwers MC, Finelli A, Campbell CM, Marlow BA, Silver IL. Physician self-audit: a scoping review. *Journal of Continuing Education in the Health Professions.* 2011; 31(4):258-264.

169. Gagliardi AR, Fenech D, Eskicioglu C, Nathens AB, McLeod R. Factors influencing antibiotic prophylaxis for surgical site infection prevention in general surgery: a review of the literature. *Canadian Journal of Surgery.* 2009; 52(6):481-489.

170. Galloway T, Blackett H, Chatwood S, Jeppesen C, Kandola K, Linton J *et al*. Obesity studies in the circumpolar Inuit: a scoping review. *International journal of circumpolar health.* 2012; 71.

171. Ganann R, Fitzpatrick-Lewis D, Ciliska D, Peirson L. Community-based interventions for enhancing access to or consumption of fruit and vegetables among five to 18-year olds: a scoping review. *BMC Public Health.* 2012; 12(711).

172. Gangapersad J, Brouwer A, Kurilsky S, Willis E, Shaw L. A scoping review of the knowledge base in WORK that addresses work related outcomes for individuals with chronic pain. *Work.* 2010; 35(3):283-299.

173. Garcia-Sanjuan S, Lillo-Crespo M, Sanjuan-Quiles A, Gil-Gonzalez D, Richart-Martinez M. Life Experiences of People Affected by Crohn's Disease and Their Support Networks: Scoping Review. *Clin Nurs Res.* 2014:1-21.

174. Gardois P, Colombi N, Grillo G, Villanacci MC. Implementation of Web 2.0 services in academic, medical and research libraries: a scoping review. *Health Information and Libraries Journal.* 2012; 29(2):90-109.

175. Gentles SJ, Lokker C, McKibbon KA. Health information technology to facilitate communication involving health care providers, caregivers, and pediatric patients: a scoping review. *Journal of Medical Internet Research.* 2010; 12(2):e22.

176. Glegg SM, Tatla SK, Holsti L. The GestureTek virtual reality system in rehabilitation: a scoping review. *Disability and Rehabilitation: Assistive Technology.* 2014; 9(2):89-111.

177. Godfrey CM, Harrison MB, Lang A, Macdonald M, Leung T, Swab M. Homecare safety and medication management with older adults: A scoping review of the quantitative and qualitative evidence. *JBI Database of Systematic Reviews and Implementation Reports.* 2013; 11(7):82-130.

178. Goldman J, Zwarenstein M, Bhattacharyya O, Reeves S. Improving the clarity of the interprofessional field: implications for research and continuing interprofessional education. *Journal of Continuing Education in the Health Professions.* 2009; 29(3):151-156.

179. Goldner EM, Jeffries V, Bilsker D, Jenkins E, Menear M, Petermann L. Knowledge translation in mental health: a scoping review. *Healthcare Policy.* 2011; 7(2):83-98.

180. Goldstein DP, Ringash J, Bissada E, Jaquet Y, Irish J, Chepeha D *et al*. Scoping review of the literature on shoulder impairments and disability after neck dissection. *Head & Neck.* 2014; 36(2):299-308.

181. Gonzalez MT, Kirkevold M. Benefits of sensory garden and horticultural activities in dementia care: a modified scoping review. *J Clin Nurs.* 2014; 23(19-20):2698-2715.

182. Goodridge D, Hawranik P, Duncan V, Turner H. Socioeconomic disparities in home health care service access and utilization: a scoping review. *International Journal of Nursing Studies.* 2012; 49(10):1310-1319.

183. Gordon AL, Logan PA, Jones RG, Forrester-Paton C, Mamo JP, Gladman JR *et al*. A systematic mapping review of randomized controlled trials (RCTs) in care homes. *BMC Geriatrics.* 2012; 12(31).

184. Goryakin Y, Griffiths P, Maben J. Economic evaluation of nurse staffing and nurse substitution in health care: a scoping review. *International Journal of Nursing Studies.* 2011; 48(4):501-512.

185. Gowing LR, Ali RL, White JM. Systematic review processes and the management of opioid withdrawal. *Australian and New Zealand Journal of Public Health.* 2000; 24(4):427-431.

186. Grabovschi C, Loignon C, Fortin M. Mapping the concept of vulnerability related to health care disparities: a scoping review. *BMC Health Services Research* 2013; 13(94).

187. Gray L, Ng H, Bartlett D. The gross motor function classification system: an update on impact and clinical utility. *Pediatric physical therapy.* 2010; 22(3):315-320.

188. Greer N, Brasure M, Wilt T. Wheeled Mobility (Wheelchair) Service Delivery: Scope of the Evidence. *Annals of Internal Medicine.* 2012; 156(2):141-146.

189. Greyson DL, Becu AR, Morgan SG. Sex, drugs and gender roles: mapping the use of sex and gender based analysis in pharmaceutical policy research. *Int J Equity Health.* 2010; 9:26.

190. Gridley K, Brooks J, Glendinning C. Good practice in social care for disabled adults and older people with severe and complex needs: evidence from a scoping review. *Health and Social Care in the community.* 2014; 22(3):234-248.

191. Griffiths P, Bennett J, Smith E. The research base for learning disability nursing: A rapid scoping review. In*.*: King's College London; 2007.

192. Griffiths P, Bennett J, Smith E. The size, extent and nature of the learning disability nursing research base: A systematic scoping review. *International journal of nursing studies.* 2009; 46(4):490-507.

193. Griffiths P, Renz A, Hughes J, Rafferty A. Impact of organisation and management factors on infection control in hospitals: a scoping review. *Journal of Hospital Infection.* 2009; 73(1):1-14.

194. Griffiths P, Richardson A, Blackwell R. Outcomes sensitive to nursing service quality in ambulatory cancer chemotherapy: systematic scoping review. *European Journal of Oncology Nursing.* 2012; 16(3):238-246.

195. Grindrod K, Forgione A, Tsuyuki RT, Gavura S, Giustini D. Pharmacy 2.0: a scoping review of social media use in pharmacy. *Research in Social and Administrative Pharmacy.* 2014; 10(1):256-270.

196. Guilcher SJ, Craven BC, McColl MA, Lemieux-Charles L, Casciaro T, Jaglal SB. Application of the Andersen's health care utilization framework to secondary complications of spinal cord injury: a scoping review. *Disability and Rehabilitation.* 2012; 34(7):531-541.

197. Gulliford M, Morgan M, Hughes D, Beech R, Figeroa-Munoz J, Gibson B *et al*. Access to health care. Report of a scoping exercise for the National Co-ordinating Centre for NHS Service Delivery and Organisation R&D (NCCSDO). In*.* London; 2001.

198. Gussy M, Dickson‐Swift V, Adams J. A scoping review of qualitative research in peer‐reviewed dental publications. *International journal of dental hygiene.* 2013; 11(3):174-179.

199. Gysels M, Evans N, Menaca A, Andrew E, Toscani F, Finetti S *et al*. Culture and end of life care: a scoping exercise in seven European countries. *PLoS One.* 2012; 7(4):e34188.

200. Hagell A, Dowling SB. Scoping review of literature on the health and care of mentally disordered offenders, vol. 16. York: University of York; 1999.

201. Hamm MP: Knowledge Translation to Improve Research and Decision-making in Child Health. *Thesis.* University of Alberta; 2013.

202. Hamm MP, Chisholm A, Shulhan J, Milne A, Scott SD, Given LM *et al*. Social media use among patients and caregivers: a scoping review. *BMJ open.* 2013; 3(5):e002819.

203. Han CS, Ogrodniczuk JS, Oliffe JL. Qualitative research on suicide in East Asia: A scoping review. *Journal of Mental Health.* 2013; 22(4):372-383.

204. Han CS, Oliffe JL, Ogrodniczuk JS. Suicide among East Asians in North America: a scoping review. *Journal of Mental Health.* 2013; 22(4):361-371.

205. Hand C, Law M, McColl MA. Occupational therapy interventions for chronic diseases: a scoping review. *American Journal of Occupational Therapy.* 2011; 65(4):428-436.

206. Hand C, Law MC, McColl MA, Hanna S, Elliott SJ. Neighborhood influences on participation among older adults with chronic health conditions: A scoping review. *OTJR: Occupation, Participation and Health.* 2012; 32(3):95-109.

207. Hand C, Letts L. Occupational therapy research and practice involving adults with chronic diseases: A scoping review and internet scan. In*.* Ottawa; 2009.

208. Hanson EK, Beukelman DR, Yorkston KM. Communication support through multimodal supplementation: a scoping review. *Augmentative and Alternative Communication.* 2013; 29(4):310-321.

209. Harden A, Josephine K, Powell C, Oliver K, Oakley A. A scoping review of the evidence relevant to life checks for young people aged 9 to 14 years. In*.*: EPPI-Centre, Social Science Research Unit, Institute of Education, University of London; 2007.

210. Harris J, Kroll T, Law J, Bolik F. Disability and homelessness in Central and Northern Scotland: Interdisciplinary Disability Research Institute; 2006.

211. Harris M, Rhodes T. Hepatitis C treatment access and uptake for people who inject drugs: a review mapping the role of social factors. *Harm Reduction Journal.* 2013; 10(1):7.

212. Harrison MB, Keeping-Burke L, Godfrey CM, Ross-White A, McVeety J, Donaldson V *et al*. Safety in home care: A mapping review of the international literature. *International Journal of Evidence-Based Healthcare.* 2013; 11(3):148-160.

213. Hartman LR, Magalhaes L, Mandich A. What does parental divorce or marital separation mean for adolescents? A scoping review of North American literature. *Journal of Divorce and Remarriage.* 2011; 52(7):490-518.

214. Heller T, Fisher D, Marks B, Hsieh K. Interventions to promote health: crossing networks of intellectual and developmental disabilities and aging. *Disability and Health Journal.* 2014; 7(1 Suppl):S24-S32.

215. Heller T, McCubbin JA, Drum C, Peterson J. Physical activity and nutrition health promotion interventions: what is working for people with intellectual disabilities? *Intellectual and Developmental Disabilities.* 2011; 49(1):26-36.

216. Hempel S, Chambers D, Bagnall A-M, Forbes C. Risk factors for chronic fatigue syndrome/myalgic encephalomyelitis: a systematic scoping review of multiple predictor studies. *Psychological medicine.* 2008; 38(07):915-926.

217. Hempel S, Norman G, Golder S, Aguiar-Ibáñez R, Eastwood A. Psychosocial interventions for non-professional carers of people with Parkinson's Disease: a scoping review. York: University of York; 2007.

218. Hempel S, Norman G, Golder S, Aguiar‐Ibáñez R, Eastwood A. Psychosocial interventions for non‐professional carers of people with Parkinson’s disease: a systematic scoping review. *Journal of advanced nursing.* 2008; 64(3):214-228.

219. Hewitt C, Lankshear A, Kazanjian A, Maynard A, Sheldon T, Smith K. Health Service Workforce and Health Outcomes: A Scoping Study. In*.*; 2005.

220. Higginson IJ, Shipman C, Gysels M, White P, Barclay S, Forrest S *et al*. Scoping exercise on generalist services for adults at the end of life: research, knowledge, policy and future research needs. In*.*; 2007.

221. Hoare C, Li Wan PA, Williams H. Systematic review of treatments for atopic eczema. *Health Technology Assessment.* 2000; 4(37):1-191.

222. Hooker L, Ward B, Verrinder G. Domestic violence screening in maternal and child health nursing practice: a scoping review. *Contemporary Nurse.* 2012; 42(2):198-215.

223. Hooper B, King R, Wood W, Bilics A, Gupta J. An international systematic mapping review of educational approaches and teaching methods in occupational therapy. *The British Journal of Occupational Therapy.* 2013; 76(1):9-22.

224. Horne R, Weinman J, Barber N, Elliott R, Morgan M. Concordance, adherence and compliance in medicine taking. In*.*; 2005.

225. Hosking J, Campbell-Lendrum D. How well does climate change and human health research match the demands of policymakers? A scoping review. *Environmental Health Perspectives.* 2012; 120(8):1076-1082.

226. Howell D, Fitch M, Bakker D, Green E, Sussman J, Mayo S *et al*. Core domains for a person-focused outcome measurement system in cancer (PROMS-Cancer Core) for routine care: a scoping review and Canadian Delphi Consensus. *Value in Health.* 2013; 16(1):76-87.

227. Hundley VA, Avan BI, Braunholtz D, Fitzmaurice AE, Graham WJ. Lessons regarding the use of birth kits in low resource countries. *Midwifery.* 2011; 27(6):e222-e230.

228. Hundley VA, Avan BI, Braunholtz D, Graham WJ. Are birth kits a good idea? A systematic review of the evidence. *Midwifery.* 2012; 28(2):204-215.

229. Hunt AW, Turner GR, Polatajko H, Bottari C, Dawson DR. Executive function, self-regulation and attribution in acquired brain injury: A scoping review. *Neuropsychological rehabilitation.* 2013; 23(6):914-932.

230. Hunter KF, Bharmal A, Moore KN. Long-term bladder drainage: Suprapubic catheter versus other methods: a scoping review. *Neurourology and Urodynamics.* 2013; 32(7):944-951.

231. Hunter KF, Wagg A, Kerridge T, Chick H, Chambers T. Falls risk reduction and treatment of overactive bladder symptoms with antimuscarinic agents: a scoping review. *Neurourology and Urodynamics.* 2011; 30(4):490-494.

232. Hurlock-Chorostecki C, Forchuk C, Orchard C, van Soeren M, Reeves S. Hospital-based nurse practitioner roles and interprofessional practice: a scoping review. *Nurs Health Sci.* 2014; 16:403-410.

233. Hussein S, Manthorpe J, Stevens M. People in places: A qualitative exploration of recruitment agencies' perspectives on the employment of international social workers in the UK. *British Journal of Social Work.* 2010; 40(3):1000-1016.

234. Iafolla MA, Tay J, Allan DS. Transplantation of umbilical cord blood-derived cells for novel indications in regenerative therapy or immune modulation: a scoping review of clinical studies. *Biology of Blood and Marrow Transplantation.* 2014; 20(1):20-25.

235. Idzerda L, Rader T, Tugwell P, Boers M. Can we decide which outcomes should be measured in every clinical trial? A scoping review of the existing conceptual frameworks and processes to develop core outcome sets. *Journal of Rheumatology.* 2014; 41(5):986-993.

236. Ilic S, Rajić A, Britton CJ, Grasso E, Wilkins W, Totton S *et al*. A scoping study characterizing prevalence, risk factor and intervention research, published between 1990 and 2010, for microbial hazards in leafy green vegetables. *Food Control.* 2012; 23(1):7-19.

237. Istomina N, Suominen T, Razbadauskas A, Leino-Kilpi H. Research on the quality of abdominal surgical nursing care: a scoping review. *Medicina (Kaunas).* 2011; 47(5):245-256.

238. Jaana M, Vartak S, Ward MM. Evidence-based health care management: what is the research evidence available for health care managers? *Evaluation and the Health Professions.* 2014; 37(3):314-334.

239. Jacob S, Schiffino N. Risk Policies in the United States: Definition and Characteristics Based on a Scoping Review of the Literature. *Risk Anal.* 2015; 35(5):849-858.

240. Jansen YJ, Foets MM, de Bont AA. The contribution of qualitative research to the development of tailor-made community-based interventions in primary care: a review. *European Journal of Public Health.* 2009; 20(2):220-226.

241. Jbilou J, Halilem N, Blouin-Bougie J, Amara N, Landry R, Simard J. Medical genetic counseling for breast cancer in primary care: a synthesis of major determinants of physicians' practices in primary care settings. *Public Health Genomics.* 2014; 17(4):190-208.

242. Jenkins E, Goldner EM. Approaches to understanding and addressing treatment-resistant depression: a scoping review. *Depression research and treatment.* 2012; 2012.

243. Jensen MP, Truitt AR, Schomer KG, Yorkston KM, Baylor C, Molton IR. Frequency and age effects of secondary health conditions in individuals with spinal cord injury: a scoping review. *Spinal Cord.* 2013; 51(12):882-892.

244. Jepson R, Di Blasi Z, Wright K, Ter Riet G. Scoping review of the effectiveness of mental health services, vol. 21. York: University of York; 2001.

245. Jiancaro T, Jamieson GA, Mihailidis A. Twenty years of cognitive work analysis in health care: A scoping review. *Journal of Cognitive Engineering and Decision Making.* 2014; 8(1):3-22.

246. Johnston R, Crooks VA, Snyder J, Kingsbury P. What is known about the effects of medical tourism in destination and departure countries? A scoping review. *International Journal for Equity in Health.* 2010; 9:24.

247. Jones CA, Pohar S. Health-related quality of life after total joint arthroplasty: a scoping review. *Clinics in Geriatric Medicine.* 2012; 28(3):395-429.

248. Jones R, Everson-Hock ES, Papaioannou D, Guillaume L, Goyder E, Chilcott J *et al*. Factors associated with outcomes for looked-after children and young people: a correlates review of the literature. *Child: Care, Health and Development.* 2011; 37(5):613-622.

249. Jowsey T, Yen L, Pm W. Time spent on health related activities associated with chronic illness: a scoping literature review. *BMC Public Health.* 2012; 12(1044).

250. Joy SM, Little E, Maruthur NM, Purnell TS, Bridges JF. Patient preferences for the treatment of type 2 diabetes: a scoping review. *PharmacoEconomics.* 2013; 31(10):877-892.

251. Kania A, Patel AB, Roy A, Yelland GS, Nguyen DTK, Verhoef MJ. Capturing the complexity of evaluations of health promotion interventions: A scoping review. *Canadian Journal of Program Evaluation.* 2013; 27(1):65-91.

252. Karsh J, Keystone EC, Haraoui B, Thorne JC, Pope JE, Bykerk VP *et al*. Canadian recommendations for clinical trials of pharmacologic interventions in rheumatoid arthritis: inclusion criteria and study design. *Journal of Rheumatology.* 2011; 38(10):2095-2104.

253. Katz DL, Williams AL, Girard C, Goodman J, Comerford B, Behrman A *et al*. The evidence base for complementary and alternative medicine: methods of Evidence Mapping with application to CAM. *Altern Ther Health Med.* 2003; 9(4):22-30.

254. Kavanagh J, Stansfield C, Thomas J. Incentives to improve smoking, physical activity, dietary and weight management behaviours: A scoping review of the research evidence. In*.*: EPPI Centre, Social Science Research Unit, Institute of Education, University of London; 2009.

255. Kavanagh J, Trouton A, Oakley A, Harden A. A scoping review of the evidence for incentive schemes to encourage positive health and other social behaviours in young people. In*.* London: EPPI-Centre, Social Science Research Unit, Institute of Education, University of London; 2005.

256. Keightley ML, Chen JK, Ptito A. Examining the neural impact of pediatric concussion: a scoping review of multimodal and integrative approaches using functional and structural MRI techniques. *Current Opinion in Pediatrics.* 2012; 24(6):709-716.

257. Keightley ML, Sinopoli KJ, Davis KD, Mikulis DJ, Wennberg R, Tartaglia MC *et al*. Is there evidence for neurodegenerative change following traumatic brain injury in children and youth? A scoping review. *Frontiers in Human Neuroscience.* 2014.

258. Kelleher LK, Campbell KR, Dickey JP. Biomechanical research on bowed string musicians: a scoping study. *Medical Problems of Performing Arts.* 2013; 28(4):212-218.

259. Kembhavi G, Darrah J, Payne K, Plesuk D. Adults with a diagnosis of cerebral palsy: a mapping review of long-term outcomes. *Developmental Medicine and Child Neurology.* 2011; 53(7):610-614.

260. Kendall S, Wilson P, Procter S, Brooks F, Bunn F, Gage H *et al*. The nursing contribution to chronic disease management: A whole systems approach. In*.*; 2010.

261. Kenny A, Hyett N, Sawtell J, Dickson-Swift V, Farmer J, O'Meara P. Community participation in rural health: a scoping review. *BMC Health Services Research.* 2013; 13(64).

262. Kidd SA, McKenzie KJ, Virdee G. Mental health reform at a systems level: widening the lens on recovery-oriented care. *Canadian Journal of Psychiatry.* 2014; 59(5):243.

263. Kim J. Complex interventions in healthcare and health informatics: a scoping review. *Enabling Health and Healthcare Through ICT: Available, Tailored, and Closer.* 2013; 183:263.

264. Kimber M, Couturier J, Georgiades K, Wahoush O, Jack SM. Body image dissatisfaction among immigrant children and adolescents in Canada and the United States: A scoping review. *International Journal of Eating Disorders.* 2014; 47(8):892-897.

265. King G, Currie M, Petersen P. Child and parent engagement in the mental health intervention process: a motivational framework. *Child and Adolescent Mental Health.* 2014; 19(1):2-8.

266. King JL, Pomeranz JL, Merten JW. Nutrition interventions for people with disabilities: A scoping review. *Disability and Health Journal.* 2014; 7(2):157-163.

267. Kinnunen U-M, Sarantob K. It is time for self-incident-reporting for patients and their families in every health care organization: a literature review. *Studies in health technology and informatics.* 2012; 192:92-96.

268. Kirk SF, Penney TL, McHugh TL. Characterizing the obesogenic environment: the state of the evidence with directions for future research. *Obesity Reviews.* 2010; 11(2):109-117.

269. Kirst M, Zhang YJ, Young A, Marshall A, O’Campo P, Ahmad F. Referral to Health and Social Services for Intimate Partner Violence in Health Care Settings A Realist Scoping Review. *Trauma, Violence, & Abuse.* 2012; 13(4):198-208.

270. Klinger CA, Howell D, Zakus D, Deber RB. Barriers and facilitators to care for the terminally ill: a cross-country case comparison study of Canada, England, Germany, and the United States. *Palliative Medicine.* 2013; 28(2):111-120.

271. Knight R, Shoveller J, Greyson D, Kerr T, Gilbert M, Shannon K. Advancing population and public health ethics regarding HIV testing: A scoping review. *Critical Public Health.* 2014(3):283-295.

272. Knight R, Small W, Pakula B, Thomson K, Shoveller J. A scoping study to identify opportunities to advance the ethical implementation and scale-up of HIV treatment as prevention: priorities for empirical research. *BMC Medical Ethics.* 2014; 15(54).

273. Koch S, Vimarlund V. Critical advances in bridging personal health informatics and clinical informatics. *Yearbook of medical informatics.* 2012; 7(1):48-55.

274. Koehlmoos T, Gazi R, Hossain S, Rashid M. Social franchising evaluations: a scoping review. In*.*; 2011.

275. Koehn S, Neysmith S, Kobayashi K, Khamisa H. Revealing the shape of knowledge using an intersectionality lens: Results of a scoping review on the health and health care of ethnocultural minority older adults. *Ageing and Society.* 2013; 33(3):437-464.

276. Koskinen S, Salminen L, Stolt M, Leino‐Kilpi H. The education received by nursing students regarding nursing older people: a scoping literature review. *Scandinavian journal of caring sciences.* 2014; 29(1):15-29.

277. Kovacs BK, Bellows M, Eigenseher C, Gallivan J. 'Practical' resources to support patient and family engagement in healthcare decisions: a scoping review. *BMC Health Services Research* 2014; 14(175).

278. Kuhlmann E, Batenburg R, Groenewegen PP, Larsen C. Bringing a European perspective to the health human resources debate: A scoping study. *Health Policy.* 2013; 110(1):6-13.

279. Kushki A, Chau T, Anagnostou E. Handwriting difficulties in children with autism spectrum disorders: a scoping review. *Journal of Autism and Developmental Disorders.* 2011; 41(12):1706-1716.

280. Kwak L, Hagstromer M, Jensen I, Karlsson ML, Alipour A, Elinder LS. Promoting physical activity and healthy dietary behavior: the role of the occupational health services: a scoping review. *Journal of Occupational and Environmental Medicine.* 2014; 56(1):35-46.

281. Lal S, Jarus T, Suto MJ. A scoping review of the Photovoice method: implications for occupational therapy research. *Canadian Journal of Occupational Therapy.* 2012; 79(3):181-190.

282. Lederer V, Loisel P, Rivard M, Champagne F. Exploring the diversity of conceptualizations of work (dis)ability: a scoping review of published definitions. *Journal of Occupational Rehabilitation.* 2014; 24(2):242-267.

283. Lee K, Kamradt-Scott A. The multiple meanings of global health governance: a call for conceptual clarity. *Global health.* 2014; 10(28):1-10.

284. Leland NE, Elliott SJ, O'Malley L, Murphy SL. Occupational therapy in fall prevention: current evidence and future directions. *American Journal of Occupational Therapy.* 2012; 66(2):149-160.

285. Leland NE, Marcione N, Niemiec SLS, Kelkar K, Fogelberg D. What is occupational therapy's role in addressing sleep problems among older adults? *OTJR: occupation, participation and health.* 2014; 34(3):141-149.

286. Levac D, Colquhoun H, O'Brien KK. Scoping studies: advancing the methodology. *Implementation Science.* 2010; 5(69).

287. Levac D, Rivard L, Missiuna C. Defining the active ingredients of interactive computer play interventions for children with neuromotor impairments: a scoping review. *Research in Developmental Disabilities.* 2012; 33(1):214-223.

288. Levac D, Wishart L, Missiuna C, Wright V. The application of motor learning strategies within functionally based interventions for children with neuromotor conditions. *Pediatric Physical Therapy.* 2009; 21(4):345-355.

289. Levasseur M, Carrier A. Integrating health literacy into occupational therapy: findings from a scoping review. *Scandinavian journal of occupational therapy.* 2012; 19(4):305-314.

290. Levesque J-F, Breton M, Senn N, Levesque P, Bergeron P, Roy DA. The Interaction of Public Health and Primary Care: Functional Roles and Organizational Models that Bridge Individual and Population Perspectives. *Public Health Reviews.* 2013; 35(1):1-27.

291. Liddy C, Johnston S, Irving H, Nash K. The Community Connection Model: implementation of best evidence into practice for self-management of chronic diseases. *Public Health.* 2013; 127(6):538-545.

292. Liu P, Parker AG, Hetrick SE, Callahan P, de Silva S, Purcell R. An evidence map of interventions across premorbid, ultra-high risk and first episode phases of psychosis. *Schizophr Res.* 2010; 123(1):37-44.

293. Livingstone R, Paleg G. Practice considerations for the introduction and use of power mobility for children. *Developmental Medicine and Child Neurology.* 2013; 56(3):210-221.

294. Lord P, Springate I, Atkinson M, Haines B, Morris M, O'Donnell L *et al*. Improving development outcomes for children through effective practice in integrating early years services (C4EO Scoping Review 1). In*.*; 2008.

295. Loutfy MR, Sherr L, Sonnenberg-Schwan U, Walmsley SL, Johnson M, d'Arminio MA *et al*. Caring for women living with HIV: gaps in the evidence. *Journal of the International AIDS Society.* 2013; 16(18509).

296. Lovell K, Bee P. Optimising treatment resources for OCD: a review of the evidence base for technology-enhanced delivery. *Journal of Mental Health.* 2011; 20(6):525-542.

297. Lunsky Y, Tint A, Robinson S, Gordeyko M, Ouellette-Kuntz H. System-wide information about family carers of adults with intellectual/developmental disabilities-A scoping review of the literature. *Journal of Policy and Practice in Intellectual Disabilities.* 2014; 11(1):8-18.

298. Lysaght R, Cobigo V, Hamilton K. Inclusion as a focus of employment-related research in intellectual disability from 2000 to 2010: a scoping review. *Disability and rehabilitation.* 2012; 34(16):1339-1350.

299. MacDonald JA, Edwards N, Davies B, Marck P, Guernsey JR. Priority setting and policy advocacy by nursing associations: a scoping review and implications using a socio-ecological whole systems lens. *Health Policy.* 2012; 107(1):31-43.

300. Macdonald M, Lang A. Applying Risk Society Theory to findings of a scoping review on caregiver safety. *Health and Social Care in the Community.* 2014; 22(2):124-133.

301. Macdonald M, Lang A, Storch J, Stevenson L, Donaldson S, Barber T *et al*. Home care safety markers: a scoping review. *Home Health Care Services Quarterly.* 2013; 32(2):126-148.

302. Macdonald MT, Lang A, Storch J, Stevenson L, Barber T, Iaboni K *et al*. Examining markers of safety in homecare using the international classification for patient safety. *BMC Health Services Research* 2013; 13(191).

303. MacDougall A, Cobban S, Compton S. Is periodontal disease related to adverse pregnancy outcomes? A scoping review. *Canadian Journal of Dental Hygiene.* 2011; 45(1):53-60.

304. MacEntee MI, Kazanjian A, Kozak JF, Hornby K, Thorne S, Kettratad-Pruksapong M. A scoping review and research synthesis on financing and regulating oral care in long-term care facilities. *Gerodontology.* 2012; 29(2):e41-e52.

305. Macpherson K. Screening hips of newborns in Scotland: A Health Technology Assessment scoping report. In*.*: NHS Quality Improvement Scotland; 2006: 34.

306. Magalhaes L, Carrasco C, Gastaldo D. Undocumented migrants in Canada: a scope literature review on health, access to services, and working conditions. *Journal of Immigrant and Minority Health.* 2010; 12(1):132-151.

307. Mala A, Karkou V, Meekums B. Dance/Movement Therapy (D/MT) for depression: A scoping review. *The Arts in Psychotherapy.* 2012; 39(4):287-295.

308. Malachowski C, Kirsh B. Workplace antistigma initiatives: a scoping study. *Psychiatric Services.* 2013; 64(7):694-702.

309. Manthorpe J, Livsey L. European challenges in delivering social services in rural regionses regions rurales de l'Europe: une etude des recherches. *European Journal of Social Work.* 2009; 12(1):5-24.

310. Manthorpe J, Martineau S. Scoping review of the research and evidence base relating to advocacy services and older people’s entry into care homes in England. In*.*; 2009.

311. Manthorpe J, Martineau S. Deciding to move to a care home: The shared territory of advocacy and social work support. *Practice: Social Work in Action.* 2010; 22(4):217-231.

312. Manthorpe J, Martineau S, Moriarty J, Hussein S, Stevens M. Support workers in social care in England: a scoping study. *Health and Social Care in the Community.* 2010; 18(3):316-324.

313. Manthorpe J, Moriarty J. Examining day centre provision for older people in the UK using the Equality Act 2010: findings of a scoping review. *Health and Social Care in the Community.* 2014; 22(4):352-360.

314. Manthorpe J, Moriarty J, Cornes M. Keeping it in the family? People with learning disabilities and families employing their own care and support workers: findings from a scoping review of the literature. *Journal of Intellectual Disabilities.* 2011; 15(3):195-207.

315. Manthorpe J, Samsi K. Improving practice in communication with older people and support networks living in housing with care schemes: Aspirations and ambitions. *British Journal of Social Work.* 2012; 42(8):1495-1512.

316. Marsella A. Exploring the literature surrounding the transition into palliative care: a scoping review. *International Journal of Palliative Nursing.* 2009; 15(4):186-189.

317. Martin K, Wilkin A, Morris M, O’Donnell L, Sharp C. Improving the wellbeing of disabled children through early years interventions (age 0–8) (C4EO Scoping Review 1). In*.*; 2009.

318. Martin-Misener R, Valaitis R, Wong ST, Macdonald M, Meagher-Stewart D, Kaczorowski J *et al*. A scoping literature review of collaboration between primary care and public health. *Primary Health Care Research & Development.* 2012; 13(4):327-346.

319. Mason A. Support services for carers of people with mental health problems: building an evidence base to inform policy decisions. *Applied Health Economics and Health Policy.* 2003; 2(1):37-42.

320. Masotti P, McColl MA, Green M. Adverse events experienced by homecare patients: a scoping review of the literature. *International Journal for Quality in Health Care.* 2010; 22(2):115-125.

321. McAloney K, Graham H, Law C, Platt L. A scoping review of statistical approaches to the analysis of multiple health-related behaviours. *Preventive Medicine.* 2013; 56(6):365-371.

322. McColl MA. Postacute programming for community integration: A scoping review. *Brain Impairment.* 2007; 8(3):238-250.

323. McColl MA, Aiken A, McColl A, Sakakibara B, Smith K. Primary care of people with spinal cord injury: scoping review. *Canadian Family Physician.* 2012; 58(11):1207-1216.

324. McColl MA, Law M. Interventions affecting self-care, productivity, and leisure among adults: a scoping review. *OTJR: Occupation, Participation and Health.* 2013; 33(2):110-119.

325. McColl MA, Shortt S, Godwin M, Smith K, Rowe K, O'Brien P *et al*. Models for integrating rehabilitation and primary care: a scoping study. *Archives of Physical Medicine and Rehabilitation.* 2009; 90(9):1523-1531.

326. McCormack A, Fortnum H. Why do people fitted with hearing aids not wear them? *International Journal of Audiology.* 2013; 52(5):360-368.

327. McCormack A, Griffiths MD. A scoping study of the structural and situational characteristics of internet gambling. *International Journal of Cyber Behavior, Psychology and Learning.* 2013; 3(1):29-49.

328. McGrath CE, Rudman DL. Factors that influence the occupational engagement of older adults with low vision: A scoping review. *The British Journal of Occupational Therapy.* 2013; 76(5):234-241.

329. McIntyre A, Janzen S, Teasell R. Traumatic brain injury in older adults: A review. *Topics in Geriatric Rehabilitation.* 2014; 30(3):230-236.

330. McKellar KA, Pitzul KB, Yi JY, Cole DC. Evaluating communities of practice and knowledge networks: a systematic scoping review of evaluation frameworks. *Ecohealth.* 2014; 11(3):383-399.

331. McKinstry C, Brown T, Gustafsson L. Scoping reviews in occupational therapy: the what, why, and how to. *Australian Occupational Therapy Journal.* 2014; 61(2):58-66.

332. McLinden T, Sargeant JM, Thomas MK, Papadopoulos A, Fazil A. Component costs of foodborne illness: a scoping review. *BMC Public Health.* 2014; 14(509).

333. McMullen SM, Meade M, Rose L, Burns K, Mehta S, Doyle R *et al*. Partial ventilatory support modalities in acute lung injury and acute respiratory distress syndrome-a systematic review. *PLoS One.* 2012; 7(8):e40190, 42012.

334. McVicar A, Munn-Giddings C, Seebohm P. Workplace stress interventions using participatory action research designs. *International Journal of Workplace Health Management.* 2013; 6(1):18-37.

335. Medves J, Van Dijk J, Edgelow M, Saxe-Braithwaite M. Scoping review of pre-registration literature on curricula for interprofessional education. Kingston (ON): HealthForce Ontario: The Interprofessional Care Strategic Implementation Committee; 2009.

336. Mickan S, Tilson JK, Atherton H, Roberts NW, Heneghan C. Evidence of effectiveness of health care professionals using handheld computers: a scoping review of systematic reviews. *Journal of Medical Internet Research.* 2013; 15(10):e212, 2013.

337. Min B, Allen-Scott LK, Buntain B. Transdisciplinary research for complex One Health issues: a scoping review of key concepts. *Preventive Veterinary Medicine.* 2013; 112(3-4):222-229.

338. Mitchell W, Glendinning C. Risk and adult social care: Identification, management and new policies. What does UK research evidence tell us? *Health, Risk and Society.* 2008; 10(3):297-315.

339. Mitton C, Smith N, Peacock S, Evoy B, Abelson J. Public participation in health care priority setting: A scoping review. *Health Policy.* 2009; 91(3):219-228.

340. Monkman H, Borycki EM, Kushniruk AW, Kuo MH. Exploring the contextual and human factors of electronic medication reconciliation research: a scoping review. *Studies in Health Technology and Informatics.* 2013; 194:166.

341. Moore L, Stelfox HT, Boutin A, Turgeon AF. Trauma center performance indicators for nonfatal outcomes: a scoping review of the literature. *Journal of Trauma and Acute Care Surgery.* 2013; 74(5):1331-1343.

342. Moriarty J, Manthorpe J. Shared expectations? Reforming the social work qualifying curriculum in England. *Social Work Education.* 2013; 32(7):841-853.

343. Moriarty J, Manthorpe J. Post-qualifying education for social workers: A continuing problem or a new opportunity? *Social Work Education.* 2014; 33(3):397-411.

344. Moriarty J, Manthorpe J, Stevens M, Hussein S. Making the transition: Comparing research on newly qualified social workers with other professions. *British Journal of Social Work.* 2011; 41(7):1340-1356.

345. Mountain GA. Self-management for people with early dementia: An exploration of concepts and supporting evidence. *Dementia: The International Journal of Social Research and Practice.* 2006; 5(3):429-446.

346. Murray SF, Pearson SC. Maternity referral systems in developing countries: current knowledge and future research needs. *Social Science and Medicine.* 2006; 62(9):2205-2215.

347. Naik-Panvelkar P, Armour C, Saini B. Discrete choice experiments in pharmacy: a review of the literature. *International Journal of Pharmacy Practice.* 2013; 21(1):3-19.

348. Neves J, Lavis JN, Ranson MK. A scoping review about conference objectives and evaluative practices: how do we get more out of them? *Health Research Policy and Systems.* 2012; 10(26).

349. Newbigging K, Bola M, Shah A. Scoping exercise with Black and minority ethnic groups on perceptions of mental wellbeing in Scotland. In*.*: Institute for Philosophy, Diversity and Mental Health, Centre for Ethnicity and Health; 2008.

350. Nguyen L, Cobban SJ, Keenan L. Caffeine as an adjuvant to common over the counter analgesics for postoperative dental pain: A scoping review. *Canadian Journal of Dental Hygiene.* 2012; 46(1):57-62.

351. Nicolson P, Beverley C, Booth A, Burr J, Collins K, Cooper C *et al*. Eliciting users’ views of the processes of health care. In*.*: Sheffield: School of Health and Related Research; 2000.

352. Nix CM, Margarido CB, Awad IT, Avila A, Cheung JJ, Dubrowski A *et al*. A scoping review of the evidence for teaching ultrasound-guided regional anesthesia. *Regional Anesthesia and Pain Medicine.* 2013; 38(6):471-480.

353. Njelesani J, Couto S, Cameron D. Disability and rehabilitation in Tanzania: a review of the literature. *Disability and Rehabilitation.* 2011; 33(23-24):2196-2207.

354. Njelesani J, Tang A, Jonsson H, Polatajko H. Articulating an occupational perspective. *Journal of Occupational Science.* 2014; 21(2):226-235.

355. Nolte E, Roland M, Guthrie S, Brereton L. Preventing emergency readmissions to hospital: A scoping review. In*.*; 2012.

356. Northway R, Davies R, Jenkins R, Mansell I. Evidencing Good Practice in Adult Protection: Informing the Protection of People with Learning Disabilities from Abuse. *The Journal of Adult Protection.* 2005; 7(2):28-36.

357. O'Brien K, Bone G, Zack E, Solomon P. HIV and rehabilitation: development of a conceptual framework for curriculum planning. *International Journal of Rehabilitation Research.* 2008; 31(3):189-197.

358. O'Brien K, Wilkins A, Zack E, Solomon P. Scoping the field: identifying key research priorities in HIV and rehabilitation. *AIDS Behavior.* 2010; 14(2):448-458.

359. O'Cathain A, Thomas KJ, Drabble SJ, Rudolph A, Hewison J. What can qualitative research do for randomised controlled trials? A systematic mapping review. *BMJ open.* 2013; 3(6), 2013.).

360. Ogilvie I, Khoury H, Goetghebeur MM, El Khoury AC, Giaquinto C. Burden of community-acquired and nosocomial rotavirus gastroenteritis in the pediatric population of Western Europe: a scoping review. *BMC Infectious Diseases.* 2012; 12(62).

361. Olsen A, Wallace J, Maher L. Responding to Australia's National Hepatitis B Strategy 2010-13: gaps in knowledge and practice in relation to Indigenous Australians. *Australian Journal of Primary Health.* 2014; 20(2):134-142.

362. O'Malley L, Croucher K. Housing and dementia care–a scoping review of the literature. *Health & social care in the community.* 2005; 13(6):570-577.

363. O'Malley L, Croucher K. Supported Housing Services for People with Mental Health Problems: A Scoping Study. *Housing Studies.* 2005; 20(5):831-845.

364. O'Reilly GM, Cameron PA, Joshipura M. Global trauma registry mapping: a scoping review. *Injury.* 2012; 43(7):1148-1153.

365. Pagliari C, Sloan D, Gregor P, Sullivan F, Detmer D, Kahan JP *et al*. What is eHealth (4): a scoping exercise to map the field. *Journal of Medical Internet Research.* 2005; 7(1).

366. Paradis E, Leslie M, Gropper MA, Aboumatar HJ, Kitto S, Reeves S. Interprofessional care in intensive care settings and the factors that impact it: results from a scoping review of ethnographic studies. *Journal of Critical Care.* 2013; 28(6):1062-1067.

367. Paradis E, Leslie M, Puntillo K, Gropper M, Aboumatar HJ, Kitto S *et al*. Delivering interprofessional care in intensive care: a scoping review of ethnographic studies. *American Journal of Critical Care.* 2014; 23(3):230-238.

368. Parke B, Beaith A, Slater L, Clarke AM. Contextual factors influencing success or failure of emergency department interventions for cognitively impaired older people: a scoping and integrative review. *Journal of Advanced Nursing.* 2011; 67(7):1426-1448.

369. Parke B, Hunter KF, Bostrom AM, Chambers T, Manraj C. Identifying modifiable factors to improve quality for older adults in hospital: a scoping review. *Int J Older People Nurs.* 2014; 9(1):8-24.

370. Parker G, Arksey H, Harden M. Meta-review of international evidence on interventions to support carers. In*.*; 2010.

371. Parmenter L. Power and Place in the Discourse of Global Citizenship Education. *Globalisation, Societies and Education.* 2011; 9(3):14.

372. Patel P, Mitera G. A systematic scoping literature review of incorporating a total quality culture within radiotherapy staffing models: A management strategy to improve patient safety and quality of care in radiation therapy departments. *Journal of Medical Imaging and Radiation Sciences.* 2011; 42(2):81-85.

373. Paterson BL, Brewer J, Stamler LL. Engagement of parents in on-line social support interventions. *Journal of Pediatric Nursing.* 2013; 28(2):114-124.

374. Patton IT, McPherson AC. Anthropometric measurements in Canadian children: a scoping review. *Canadian Journal of Public Health.* 2013; 104(5):e369-e374.

375. Peat M, Entwistle V, Hall J, Birks Y, Golder S, Group P. Scoping review and approach to appraisal of interventions intended to involve patients in patient safety. *Journal of Health Services Research & Policy.* 2010; 15 Suppl 1:17-25.

376. Peeters JM, Wiegers TA, Friele RD. How technology in care at home affects patient self-care and self-management: a scoping review. *International journal of environmental research and public health.* 2013; 10(11):5541-5564.

377. Pham MT, Rajic A, Greig JD, Sargeant JM, Papadopoulos A, McEwen SA. A scoping review of scoping reviews: advancing the approach and enhancing the consistency. *Res Synth Methods.* 2014; 5(4):371-385.

378. Piskur B, Beurskens AJ, Jongmans MJ, Ketelaar M, Norton M, Frings CA *et al*. Parents' actions, challenges, and needs while enabling participation of children with a physical disability: a scoping review. *BMC Pediatrics* 2012; 12(177).

379. Plana I, Lavoie MA, Battaglia M, Achim AM. A meta-analysis and scoping review of social cognition performance in social phobia, posttraumatic stress disorder and other anxiety disorders. *Journal of Anxiety Disorders.* 2014; 28(2):169-177.

380. Plow MA, Finlayson M, Rezac M. A scoping review of self-management interventions for adults with multiple sclerosis. *Physical Medicine and Rehabilitation.* 2011; 3(3):251-262.

381. Pongpirul K, Robinson C. Hospital manipulations in the DRG system: A systematic scoping review. *Asian Biomedicine.* 2013; 7(3):301-310.

382. Pons-Vigues M, Diez E, Morrison J, Salas-Nicas S, Hoffmann R, Burstrom B *et al*. Social and health policies or interventions to tackle health inequalities in European cities: a scoping review. *BMC Public Health.* 2014; 14(198).

383. Population Health Improvement Research Network (PHIRN). Scoping Review of the Population Health Equity and Intervention Literature in Ontario. In*.*; 2011.

384. Porter Starr KN, McDonald SR, Bales CW. Obesity and physical frailty in older adults: a scoping review of lifestyle intervention trials. *Journal of the American Medical Directors Association.* 2014; 15(4):240-250.

385. Preaud E, Largeron N. Economic burden of non-cervical cancers attributable to human papillomavirus: a European scoping review. *Journal of Medical Economics.* 2013; 16(6):763-776.

386. Prinds C, Hvidt NC, Mogensen O, Buus N. Making existential meaning in transition to motherhood--a scoping review. *Midwifery.* 2014; 30(6):733-741.

387. Prodinger B, Magalhaes L. Advancing knowledge in work-related rehabilitation - review of research published in the journal of WORK. *Work.* 2010; 35(3):301-318.

388. Raghoonandan P, Cobban SJ, Compton SM. A scoping review of the use of fluoride varnish in elderly people living in long term care facilities. *Canadian Journal of Dental Hygiene.* 2011; 45(4):217-222.

389. Rahman S, Islam MT, Alam DS. Obesity and overweight in Bangladeshi children and adolescents: a scoping review. *BMC Public Health.* 2014; 14(70).

390. Rao DV, Warburton J, Bartlett H. Health and social needs of older Australians from culturally and linguistically diverse backgrounds: issues and implications. *Australasian Journal of Ageing.* 2006; 25(4):174-179.

391. Ravenek MJ, Bryson-Campbell MM, Shaw L, Hughes ID. Perspectives on prevention, assessment, and rehabilitation of low back pain in WORK. *Work.* 2010; 35(3):269-282.

392. Reeves S, Goldman J, Gilbert J, Tepper J, Silver I, Suter E *et al*. A scoping review to improve conceptual clarity of interprofessional interventions. *Journal of Interprofessional Care.* 2011; 25(3):167-174.

393. Reeves S, Suter E, Goldman J, Martimianakis T, Chatalalsingh C, Dematteo D. A scoping review to identify organizational and education theories relevant for interprofessional practice and education: Calgary Health Region, Queen's University Inter-Professional Patient-Centred Education Direction (QUIPPED), Canadian Interprofessional Health Collaborative (CIHC); 2007.

394. Reynolds J, Wisaijohn T, Pudpong N, Watthayu N, Dalliston A, Suphanchaimat R *et al*. A literature review: the role of the private sector in the production of nurses in India, Kenya, South Africa and Thailand. *Human Resources for Health.* 2013; 11(1):14, 2013.

395. Richardson A, Addington-Hall J, Amir Z, Foster C, Stark D, Armes J *et al*. Knowledge, ignorance and priorities for research in key areas of cancer survivorship: findings from a scoping review. *British Journal of Cancer.* 2011; 105 Suppl 1:S82-94:94.

396. Richardson A, Addington-Hall J, Stark D, Foster C, Amir Z, Sharpe M. Determining Research Priorities for Cancer Survivorship: Consultation and Evidence Review. In*.*; 2009.

397. Ridde V, Morestin F. A scoping review of the literature on the abolition of user fees in health care services in Africa. *Health Policy Plan.* 2011; 26(1):1-11.

398. Roberts GW, Irvine FE, Tranter S, Spencer LH. Identifying priorities for establishing bilingual provision in nurse education: a scoping study. *Nurse Education Today.* 2010; 30(7):623-630.

399. Rodgers M, Asaria M, Walker S, McMillan D, Lucock M, Harden M *et al*. The clinical effectiveness and cost-effectiveness of low-intensity psychological interventions for the secondary prevention of relapse after depression: a systematic review. *Health Technology Assessment.* 2012; 16(28):1-130.

400. Roland M, McDonald R, Sibbald B, Boyd A, Fotaki M, Gravelle H *et al*. Outpatient services and primary care. A scoping review of research into strategies for improving outpatient effectiveness and efficiency. In*.*: National Primary Care Research and Development Centre, Centre for Public Policy and Management, University of Manchester; 2006.

401. Rowland M, Peterson-Besse J, Dobbertin K, Walsh ES, Horner-Johnson W, Expert Panel on D *et al*. Health outcome disparities among subgroups of people with disabilities: a scoping review. *Disability and Health Journal.* 2014; 7(2):136-150.

402. Roy P, Tremblay G, Oliffe JL, Jbilou J, Robertson S. Male farmers with mental health disorders: a scoping review. *Australian Journal of Rural Health.* 2013; 21(1):3-7.

403. Samaan Z, Mbuagbaw L, Kosa D, Debono VB, Dillenburg R, Zhang S *et al*. A systematic scoping review of adherence to reporting guidelines in health care literature. *Journal of multidisciplinary healthcare.* 2013; 6:169.

404. Sanchez AL, Gabrie JA, Rueda MM, Mejia RE, Bottazzi ME, Canales M. A scoping review and prevalence analysis of soil-transmitted helminth infections in Honduras. *PLoS Neglected Tropical Diseases.* 2014; 8(1):e2653.

405. Sanmartin C, Murphy K, Choptain N, Conner-Spady B, McLaren L, Bohm E *et al*. Appropriateness of healthcare interventions: concepts and scoping of the published literature. *International Journal of Technology Assessment in Health Care.* 2008; 24(3):342-349.

406. Sanou D, O'Reilly E, Ngnie-Teta I, Batal M, Mondain N, Andrew C *et al*. Acculturation and nutritional health of immigrants in Canada: a scoping review. *Journal of Immigrant Minority Health.* 2014; 16(1):24-34.

407. Saparova D. Motivating, influencing, and persuading patients through personal health records: a scoping review. *Perspectives in Health Information Management/AHIMA, American Health Information Management Association.* 2012; 9(Summer).

408. Sarrami-Foroushani P, Travaglia J, Debono D, Braithwaite J. Key concepts in consumer and community engagement: a scoping meta-review. *BMC Health Services Research.* 2014; 14(250).

409. Saunders SL, Nedelec B. What work means to people with work disability: a scoping review. *Journal of Occupational Rehabilitation.* 2014; 24(1):100-110.

410. Sawka AM, Ismaila N, Cranney A, Thabane L, Kastner M, Gafni A *et al*. A scoping review of strategies for the prevention of hip fracture in elderly nursing home residents. *PLoS One.* 2010; 5(3):e9515.

411. Sawka AM, Naeem A, Jones J, Lowe J, Segal P, Goguen J *et al*. Persistent posttreatment fatigue in thyroid cancer survivors: a scoping review. *Endocrinology and Metabolism Clinics of North America.* 2014; 43(2):475-494.

412. Schaink AK, Kuluski K, Lyons RF, Fortin M, Jadad AR, Upshur R *et al*. A scoping review and thematic classification of patient complexity: offering a unifying framework. *Journal of Comorbidity.* 2012; 2(1):9.

413. Schwellnus H, Carnahan H. Peer-coaching with health care professionals: what is the current status of the literature and what are the key components necessary in peer-coaching? A scoping review. *Medical Teacher.* 2014; 36(1):38-46.

414. Scotland HI. What implications does the organisation of vascular services have for rates of amputation?: Technologies scoping report. In*.*; 2011.

415. Scotland HI. What is the published evidence of an association between hospital volume and outcome in elective carotid endarterectomy surgery?: Technologies scoping report. In*.*; 2011.

416. Scotland HI. What is the published evidence of an association between hospital volume and operative mortality for surgical repair (open and endovascular) of unruptured and ruptured abdominal aortic aneurysms?: Technologies scoping report. In*.*; 2011.

417. Scotland HI. Is there a difference in operative mortality between endovascular aneurysm repair and open surgery in elective abdominal aortic aneurysm?: Technologies scoping report. In*.*; 2011.

418. Scotland HI. The clinical and cost-effectiveness of radiofrequency ablation for lung cancer: Technologies scoping report. In*.*; 2012.

419. Scotland HI. What is the impact of using thresholds (both for referral and surgery) for first-eye cataract surgery on the delivery of the cataract service and the resources associated with it?: Technologies scoping report. In*.*; 2012.

420. Scotland HI. What is the relative clinical effectiveness, cost effectiveness and safety of different bariatric surgery techniques (gastric bypass, gastric banding and sleeve gastrectomy)?: Technologies scoping report. In*.*; 2012.

421. Scotland HI. In radiotherapy for cancer, what are the patient safety benefits and resource implications of the various in vivo methods of dosimetry to check received radiation dose, compared with pretreatment verification only?: Technologies scoping report. 2012.

422. Scotland HI. In patients with severe medically refractory gastroparesis (such as those requiring nutritional support), how effective and cost effective is gastric electrical stimulation (EnterraTM device) in reducing symptoms, reducing requirement for nutritional support or hospitalisation and improving quality of life, when compared with medical or alternative surgical management?: Technologies scoping report. In*.*; 2012.

423. Scott RE, Saunders C, Palacios M, Nguyen DT, Ali S: Healthy E-Health? Think ‘Environmental E-Health’! In: *Global Telehealth: Selected Papers from Global Telehealth 2010 (GT2010): 15th International Conference of the International Society for Telemedicine and EHealth and 1st National Conference of the Australasian Telehealth Society: 2010*: IOS Press; 2010: 132-138.

424. Seekins T, Shunkamolah W, Bertsche M, Cowart C, Summers JA, Reichard A *et al*. A systematic scoping review of measures of participation in disability and rehabilitation research: a preliminary report of findings. *Disability and Health Journal.* 2012; 5(4):224-232.

425. Serbinski S, Shlonsky A. Is it that we are afraid to ask? A scoping review about sons and daughters of foster parents. *Children and Youth Services Review.* 2014; 36:101-114.

426. Shachak A, Barnsley J, Tu K, Jadad AR, Lemieux-Charles L. Understanding end-user support for health information technology: a theoretical framework. *Informatics in Primary Care.* 2011; 19(3):169-172.

427. Shankardass K, Solar O, Murphy K, Greaves L, O’Campo P. A scoping review of intersectoral action for health equity involving governments. *International journal of public health.* 2012; 57(1):25-33.

428. Shanmugasegaram S, Perez-Terzic C, Jiang X, Grace SL. Cardiac rehabilitation services in low-and middle-income countries: a scoping review. *Journal of Cardiovascular Nursing.* 2014; 29(5):454-463.

429. Shareck M, Frohlich KL, Poland B. Reducing social inequities in health through settings-related interventions -- a conceptual framework. *Global Health Promotion.* 2013; 20(2):39-52.

430. Shekhawat GS, Searchfield GD, Stinear CM. Role of hearing AIDS in tinnitus intervention: a scoping review. *Journal of the American Academy of Audiology.* 2013; 24(8):747-762.

431. Shemilt I, Hollands GJ, Marteau TM, Nakamura R, Jebb SA, Kelly MP *et al*. Economic instruments for population diet and physical activity behaviour change: a systematic scoping review. *PLoS One.* 2013; 8(9):e75070.

432. Shiff NJ, Jama S, Boden C, Lix LM. Validation of administrative health data for the pediatric population: a scoping review. *BMC Health Services Research* 2014; 14(236).

433. Shrubsole C, Macmillan A, Davies M, May N. 100 Unintended consequences of policies to improve the energy efficiency of the UK housing stock. *Indoor and Built Environment.* 2014; 23(3):340-352.

434. Sims D, Cabrita Gulyurtlu SS. A scoping review of personalisation in the UK: approaches to social work and people with learning disabilities. *Health and Social Care in the Community.* 2014; 22(1):13-21.

435. Snyder J, Crooks VA, Johnston R, Kingsbury P. What do we know about Canadian involvement in medical tourism?: a scoping review. *Open Medicine.* 2011; 5(3):e139-e148.

436. South J, Meah A, Bagnall AM, Jones R. Dimensions of lay health worker programmes: results of a scoping study and production of a descriptive framework. *Global Health Promotion.* 2013; 20(1):5-15.

437. South J, Meah A, Bagnall A-M, Kinsella K, Branney P, White J *et al*. People in Public Health- a study of approaches to develop and support people in public health roles. In*.*; 2010.

438. Spilsbury K, Hewitt C, Stirk L, Bowman C. The relationship between nurse staffing and quality of care in nursing homes: a systematic review. *International Journal of Nursing Studies.* 2011; 48(6):732-750.

439. Stalker K, McArthur K. Child abuse, child protection and disabled children: A review of recent research. *Child Abuse Review.* 2012; 21(1):24-40.

440. Stathokostas L, Theou O, Little RM, Vandervoort AA, Raina P. Physical activity-related injuries in older adults: a scoping review. *Sports Medicine.* 2013; 43(10):955-963.

441. Steele CM, Cichero JA. Physiological factors related to aspiration risk: a systematic review. *Dysphagia.* 2014; 29(3):295-304.

442. Stelfox HT, Bobranska-Artiuch B, Nathens A, Straus SE. Quality indicators for evaluating trauma care: a scoping review. *Archives of Surgery.* 2010; 145(3):286-295.

443. Stepanyan K, Littlejohn A, Margaryan A. Sustainable e-Learning: Toward a Coherent Body of Knowledge. *Journal of Educational Technology and Society.* 2013; 16(2):91-102.

444. Stevens M, Kirsh B, Nixon SA. Rehabilitation interventions for children living with HIV: a scoping review. *Disability and Rehabilitation.* 2014; 36(10):865-874.

445. Stroud L, Wong BM, Hollenberg E, Levinson W. Teaching medical error disclosure to physicians-in-training: a scoping review. *Academic Medicine.* 2013; 88(6):884-892.

446. Sunderland N, Beekhuyzen J, Kendall E, Wolski M. Moving health promotion communities online: a review of the literature. *Health Information Management Journal.* 2013; 42(2):9-16.

447. Sunley R, Locke R. Exploring UK secondary teachers' professional values: an overview of the literature since 2000. *Educational research.* 2010; 52(4):409-425.

448. Suphanchaimat R, Sommanustweechai A, Khitdee C, Thaichinda C, Kantamaturapoj K, Leelahavarong P *et al*. HIV/AIDS health care challenges for cross-country migrants in low- and middle-income countries: a scoping review. *HIV/AIDS – Research and Palliative Care* 2014; 6:19-38:38.

449. Surette S, Vanderjagt L, Vohra S. Surveys of complementary and alternative medicine usage: a scoping study of the paediatric literature. *Complementary Therapies in Medicine.* 2013; 21 Suppl 1:S48-53:53.

450. Suter E, Goldman J, Martimianakis T, Chatalalsingh C, DeMatteo DJ, Reeves S. The use of systems and organizational theories in the interprofessional field: findings from a scoping review. *Journal of Interprofessional Care.* 2013; 27(1):57-64.

451. Swift KD, Sayal K, Hollis C. ADHD and transitions to adult mental health services: a scoping review. *health and development.* 2013; 40(6):775-786.

452. Symonds JE, Hagell A. Adolescents and the organisation of their school time: A review of changes over recent decades in England. *Educational Review.* 2011; 63(3):291-312.

453. Taylor RL, O'Brien L, Brown T. A scoping review of the use of elastic therapeutic tape for neck or upper extremity conditions. *Journal of Hand Therapy.* 2014; 27(3):235-246.

454. Temple B, Dube C, McMillan D, Secco L, Kepron E, Dittberner K *et al*. Pain in people with developmental disabilities: A scoping review. *Journal on Developmental Disabilities.* 2012; 18(1):73-86.

455. Templeton L, Zohhadi S, Galvani S, Velleman R. "Looking Beyond Risk": Parental Substance Misuse: Scoping Study. In*.*; 2006.

456. Terstappen V, Hanson L, McLaughlin D. Gender, Health, Labor, and Inequities: A Review of the Fair and Alternative Trade Literature. *Agriculture and Human Values.* 2013; 30(1):21-39.

457. Thomas A, Law M. Research utilization and evidence-based practice in occupational therapy: a scoping study. *American Journal of Occupational Therapy.* 2013; 67(4):e55-e65.

458. Thomas A, Menon A, Boruff J, Rodriguez AM, Ahmed S. Applications of social constructivist learning theories in knowledge translation for healthcare professionals: a scoping review. *Implementation Science.* 2014; 9(54).

459. Thompson C, Lau FY. A scoping review on health records for child-in-care. *Studies in Health Technology and Informatics.* 2013; 183.

460. Thomson L, Fayed N, Sedarous F, Ronen GM. Life quality and health in adolescents and emerging adults with epilepsy during the years of transition: a scoping review. *Developmental Medicine & Child Neurology.* 2013; 56(5):421-433.

461. Thulien NS. Innovative approaches to cervical cancer screening for sex trade workers: an international scoping review. *Journal of Obstetrics and Gynecology Canada.* 2014; 36(3):231-239.

462. Tolley JS, Foroushani PS. What do we know about one-to-one peer support for adults with a burn injury? A scoping review. *Journal of Burn Care & Research.* 2014; 35(3):233-242.

463. To-Miles F, Shaw L. Knowledge transfer with children and adolescents in promoting comfort, health, and safety in technology use: Strategies and opportunities. *Work.* 2012; 43(3):387-397.

464. Tomlinson K, Benefield P. Education and Conflict: Research and Research Possibilities. In*.*: National Foundation for Educational Research; 2005: 34.

465. Toohey AM, Rock MJ. Unleashing their potential: a critical realist scoping review of the influence of dogs on physical activity for dog-owners and non-owners. *International Journal of Behavioral Nutrition & Physical Activity.* 2011; 8:46.

466. Toto PE, Raina KD, Holm MB, Schlenk EA, Rogers JC. Best Practice physical activity programs for older adults and ADL/IADL performance: A scoping review. *Topics in Geriatric Rehabilitation.* 2013; 29(1):67-76.

467. Traynor M, Davis K, Drennan V, Goodman C, Humphrey C, Locke R *et al*. The contribution of nurse, midwife and health visitor entrepreneurs to patient choice: a scoping exercise. In*.*; 2007.

468. Trede F, McEwen C, Kenny A, O'Meara P. Supervisors' experiences of workplace supervision of nursing and paramedic students in rural settings: a scoping review. *Nurse Education Today.* 2014; 34(5):783-788.

469. Trivedi D, Brooks F, Bunn F, Graham M. Early fatherhood: a mapping of the evidence base relating to pregnancy prevention and parenting support. *Health Education Research.* 2009; 24(6):999-1028.

470. Tsulukidze M, Durand MA, Barr PJ, Mead T, Elwyn G. Providing recording of clinical consultation to patients - a highly valued but underutilized intervention: a scoping review. *Patient Education and Counseling.* 2014; 95(3):297-304.

471. Tunnicliff SA, Piercy H, Bowman CA, Hughes C, Goyder EC. The contribution of the HIV specialist nurse to HIV care: a scoping review. *Journal of Clinical Nursing.* 2013; 22(23-24):3349-3360.

472. Turner J. Building the evidence base in pre‑hospital urgent and emergency care. A review of research evidence and priorities for future research. In*.*: University of Sheffield Medical Care Research Unit; 2010.

473. Tusevljak N, Rajic A, Waddell L, Dutil L, Cernicchiaro N, Greig J *et al*. Prevalence of zoonotic bacteria in wild and farmed aquatic species and seafood: a scoping study, systematic review, and meta-analysis of published research. *Foodborne Pathog Dis.* 2012; 9(6):487-497.

474. Valaitis R, Martin-Misener R, Wong ST, Macdonald M, Meagher-Stewart D, Austin P *et al*. Methods, strategies and technologies used to conduct a scoping literature review of collaboration between primary care and public health. *Primary Health Care Research & Development.* 2012; 13(3):219-236.

475. Vallerand IA, Kalenchuk AL, McLennan JD. Behavioural treatment recommendations in clinical practice guidelines for attention-deficit/hyperactivity disorder: A scoping review. *Child and Adolescent Mental Health.* 2014.

476. van de Glind EM, van Enst WA, van Munster BC, Olde Rikkert MG, Scheltens P, Scholten RJ *et al*. Pharmacological treatment of dementia: a scoping review of systematic reviews. *Dementia and Geriatric Cognitive Disorders.* 2013; 36(3-4):211-228.

477. Van Dijk J, Medves J, Edgelow M, Saxe-Braithwaite M. Scoping Review of Post-Registration (Continuing education and post-graduate) Literature on Curricula for Interprofessional Education. In*.*; 2009.

478. Van Hout MC. Kitchen chemistry: A scoping review of the diversionary use of pharmaceuticals for non-medicinal use and home production of drug solutions. *Drug Testing and Analysis.* 2014; 6(7-8):778-787.

479. van MC, Leitz L, Scott S, Daudt H, Dennis D, Watson H *et al*. Information needs across the colorectal cancer care continuum: scoping the literature. *European Journal of Cancer Care.* 2012; 21(3):296-320.

480. Vanstone M, Hibbert K, Kinsella EA, McKenzie P, Pitman A, Lingard L. Interdisciplinary Doctoral Research Supervision: A Scoping Review. *Canadian Journal of Higher Education.* 2013; 43(2):42-67.

481. Verhoef LM, Van de Belt TH, Engelen LJ, Schoonhoven L, Kool RB. Social Media and Rating Sites as Tools to Understanding Quality of Care: A Scoping review. *Journal of Medical Internet Research.* 2014; 16(2):e56.

482. Victoor A, Delnoij DM, Friele RD, Rademakers JJ. Determinants of patient choice of healthcare providers: a scoping review. *BMC Health Services Research.* 2012; 12(272).

483. Vimarlund V, Wass S. Big data, smart homes and ambient assisted living. *Yearb.* 2014; IMIA Yearbook of Medical Informatics(1):143-149.

484. Vis SA, Strandbu A, Holtan A, Thomas N. Participation and health-A research review of child participation in planning and decision-making. *Child and Family Social Work.* 2011; 16(3):325-335.

485. Vissandjee B, Hyman I, Spitzer DL, Apale A, Kamrun N. Integration, clarification, substantiation: Sex, gender, ethnicity and migration as social determinants of women’s health. *Journal of International Women's Studies.* 2013; 8(4):32-48.

486. Wagman P. How to contribute occupationally to ecological sustainability: a literature review. *Scandinavian Journal of Occupational Therapy.* 2014; 21(3):161-165.

487. Wallace LM. Reporting Systems: a scoping study of methods of providing feedback within an organization. In: *PSRP Feeback from incident reporting systems PS028 Report to the Department of Health Patient Safety Research Programme.* 2006.

488. Watson R, Parr JR, Joyce C, May C, Le Couteur AS. Models of transitional care for young people with complex health needs: a scoping review. *Child: Care, Health and Development.* 2011; 37(6):780-791.

489. Weeks LC, Strudsholm T. A scoping review of research on complementary and alternative medicine (CAM) and the mass media: looking back, moving forward. *BMC Complementary and Alternative Medicine.* 2008; 8(43).

490. Wei AC, Urbach DR, Devitt KS, Wiebe M, Bathe OF, McLeod RS *et al*. Improving quality through process change: a scoping review of process improvement tools in cancer surgery. *BMC Surgery.* 2014; 14(45).

491. Welk B, McIntyre A, Teasell R, Potter P, Loh E. Bladder cancer in individuals with spinal cord injuries. *Spinal Cord.* 2013; 51(7):516-521.

492. While A, Forbes A, Ullman R, Murgatroyd B. The contribution of nurses, midwives and health visitors to child health and child health services: a scoping review. In*.*; 2005.

493. White DE, Straus SE, Stelfox HT, Holroyd-Leduc JM, Bell CM, Jackson K *et al*. What is the value and impact of quality and safety teams? A scoping review. *Implementation Science.* 2011; 6:97.

494. Wilhelm BJ, Rajic A, Greig J, Waddell L, Trottier G, Houde A *et al*. A systematic review/meta-analysis of primary research investigating swine, pork or pork products as a source of zoonotic hepatitis E virus. *Epidemiology and infection* 2011; 139(8):1127-1144.

495. Williams AP, Deber R, Lum J, Montgomery R, Peckham A, Kuluski K *et al*. Mapping the State of the Art: Integrating Care for Vulnerable Older. In*.*; 2009.

496. Williams C, Northstone K, Borwick C, Gainsborough M, Roe J, Howard S *et al*. How to help children with neurodevelopmental and visual problems: a scoping review. *British Journal of Ophthalmology.* 2013.

497. Williams CK, Hui Y, Borschel D, Carnahan H. A scoping review of undergraduate ambulatory care education. *Medical teacher.* 2013; 35(6):444-453.

498. Williams RM, Bambara J, Turner AP. A scoping study of one-to-one peer mentorship interventions and recommendations for application with Veterans with postdeployment syndrome. *The Journal of head trauma rehabilitation.* 2012; 27(4):261-273.

499. Williams V, Marriott A, Townsley R. Shaping our future: a scoping and consultation exercise to establish research priorities in learning disabilities for the next ten years. In*.*; 2008.

500. Williams-Brennan L, Gastaldo D, Cole DC, Paszat L. Social determinants of health associated with cervical cancer screening among women living in developing countries: a scoping review. *Archives of gynecology and obstetrics.* 2012; 286(6):1487-1505.

501. Wilson MG, Dickie M, Cooper CL, Carvalhal A, Bacon J, Rourke SB. Treatment, care and support for people co-infected with HIV and hepatitis C: a scoping review. *Open Medicine.* 2009; 3(4):e184-e195.

502. Wilson MG, Lavis JN, Guta A. Community-based organizations in the health sector: a scoping review. *Health Research Policy and Systems.* 2012; 10(36).

503. Wilson NC, Chong J, Mackey AH, Stott NS. Reported outcomes of lower limb orthopaedic surgery in children and adolescents with cerebral palsy: a mapping review. *Developmental Medicine and Child Neurology.* 2014; 56(9):808-814.

504. Wilson PM, Petticrew M, Calnan MW, Nazareth I. Disseminating research findings: what should researchers do? A systematic scoping review of conceptual frameworks. *Implementation Science.* 2010; 5(91).

505. Wise M, Angus S, Harris E, Parker S. Scoping Study of Health Promotion Tools for Aboriginal and Torres Strait Islander People. In*.*: The Lowitja Institute; 2012.

506. Wong ST, MacLeod M, Farrally V. Health human resource: scoping literature review and synthesis. In*.* Vancouver; 2009.

507. Worthington C, O'Brien K, Zack E, McKee E, Oliver B. Enhancing labour force participation for people living with HIV: a multi-perspective summary of the research evidence. *AIDS Behavior.* 2012; 16(1):231-243.

508. Wright W, McDowell JRS, Leese G, McHardy KC. A scoping exercise of work-based learning and assessment in multidisciplinary health care in Scotland. *The Journal of Practice Teaching & Learning.* 2011; 10(2):28-42.

509. Wysocki A, Butler M, Shamliyan T, Kane RL. Whole-body vibration therapy for osteoporosis: state of the science. *Ann Intern Med.* 2011; 155(10):680-686, w206-613.

510. Yost J, Thompson D, Ganann R, Aloweni F, Newman K, McKibbon A *et al*. Knowledge translation strategies for enhancing nurses' evidence-informed decision making: a scoping review. *Worldviews on Evidence-Based Nursing.* 2014; 11(3):156-167.

511. Young R, Weir H, Buchan J. Health professional mobility in Europe and the UK: a scoping study of issues and evidence. In*.*: The National Coordinating Centre for the Service Delivery and Organisation; 2010.

512. Younger P. Internet-based information-seeking behaviour amongst doctors and nurses: a short review of the literature. *Health Information and Libraries Journal.* 2010; 27(1):2-10.

513. Zaidi S, Nishtar NA. Rational prescription & use: A snapshot of the evidence from Pakistan and emerging concerns. *International Journal of Pharmacy and Pharmaceutical Sciences.* 2013; 5(Suppl1):131-135.

514. Zeigler L, Hook J, Stark D, Neilly L, Hodges L, Walker J *et al*. Systematic scope and collation of research evidence regarding interventions. In*.*; 2009.

515. Zheng Q, Vanderslott S, Jiang B, Xu LL, Liu CS, Huo LL *et al*. Research gaps for three main tropical diseases in the People's Republic of China. *Infectious Diseases of Poverty.* 2013; 2(1):15.

516. Zinszer K, Verma AD, Charland K, Brewer TF, Brownstein JS, Sun Z *et al*. A scoping review of malaria forecasting: past work and future directions. *BMJ Open.* 2012; 2(6).

# Appendix C. Word cloud of methodology cited

**
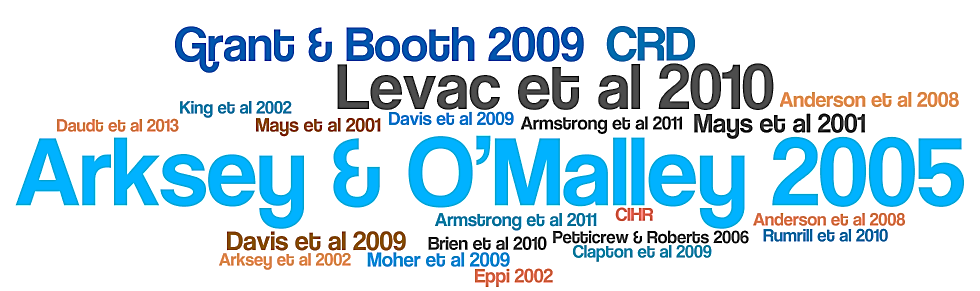
**

| **Most Cited (n=494)** | **Count (%)** |
| --- | --- |
| Arksey & O’Malley 2005 | 272 (55%) |
| Levac et al 2010 | 58 (11.7%) |
| Centre for Reviews & Dissemination | 17 (3.4%) |
| Grant & Booth 2009 | 16 (3.2%) |

# Appendix D. Purposes of conducting the scoping review

| **Research purpose (n=494)** | **Count (%)** |
| --- | --- |
| Explore breadth/extent of evidence | 336 (68%) |
| Map and summarize evidence | 177 (36%) |
| Inform future research | 103 (21%) |
| Identify knowledge gap(s) | 84 (17%) |
| Address knowledge gap(s) | 55 (11%) |
| Implications for practice and policy | 41 (8%) |
| Advance knowledge/awareness | 28 (6%) |
| Identify key themes | 22 (4%) |
| Develop a conceptual framework/map | 15 (3%) |
| Assess quality and reporting of evidence | 8 (2%) |
| Advance research methodology | 4 (1%) |
| Establish evidence base | 4 (1%) |
| Explore theories | 3 (1%) |
| Identify policy and practice gap(s) | 3 (1%) |
| Explore specific research question | 2 (0.4%) |
| Gather data for model estimates | 2 (0.4%) |
| Bibliometric analysis | 1 (0.2%) |
| Develop an evidence mapping tool | 1 (0.2%) |
| Disseminate findings | 1 (0.2%) |
| Explore emerging research area | 1 (0.2%) |
| Gather cost data | 1 (0.2%) |
| Gather data for global estimate | 1 (0.2%) |
| Inform research funding bodies | 1 (0.2%) |
| Not reported | 22(4.4%) |

*note categories are not mutually exclusive.

# Appendix E. Elements in the definition of a scoping review provided in the scoping review

| **Components listed in the conceptual definition (N=290)*** | **Count (%)** |
| --- | --- |
| Map or summarize current state of evidence [e.g. size, type(s), themes] | 242 (83%) |
| Breadth of research (targeted) | 135 (47%) |
| Identify gaps in the evidence | 107 (37%) |
| No quality assessment | 69 (24%) |
| Preliminary step to a systematic review (SR); determine feasibility of conducting a SR | 50 (17%) |
| Some similarities w/SR methods (i.e. systematic approach) | 45 (16%) |
| Novel research area that has not yet been reviewed systematically | 43 (15%) |
| Guide future research | 39 (13%) |
| A rapid process, with limits on resources, time, search strategy, etc. | 37 (13%) |
| Complex research area | 25 (9%) |
| Qualitative literature review or techniques involved (including consultation exercise) | 16 (6%) |
| Inform policy or practice | 14 (5%) |
| Iterative approach | 13 (4%) |
| *204 studies did not report a working/conceptual definition | 242 (83%) |

*note categories are not mutually exclusive.

# Appendix F. Agreement between the definition of a scoping review and the research objective(s)

# Appendix G. Types of sources searched for grey literature

| **Types of Grey Literature Searched (n = 494)** | Count (%) |
| --- | --- |
| Grey literature repository and library catalogue | 284 (57%) |
| Government websites and agency databases | 129 (26%) |
| Web search engines (i.e., Google, Google Scholar, Yahoo, Bing) | 93 (19%) |
| Conference proceedings and abstracts | 91 (18%) |
| Professional associations and organizations | 47 (10%) |
| Key non-profit organizations | 43 (9%) |
| Theses/dissertations | 30 (6%) |
| Not reported | 21 (4%) |

# Appendix H. Joanna Briggs Institute Methodology Assessment

**
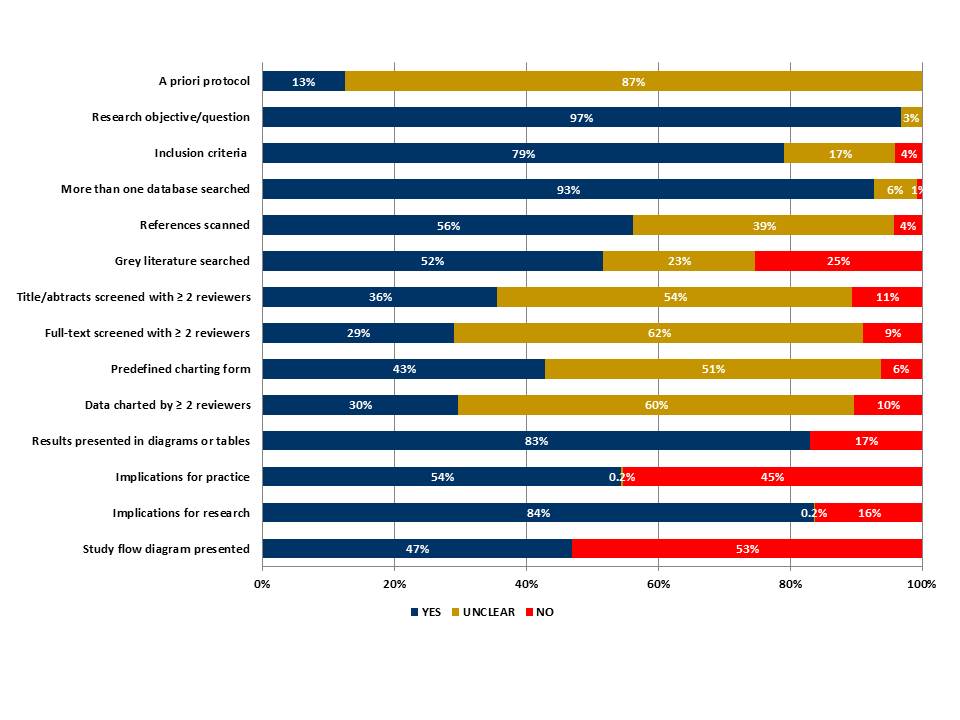
**An assessment of the methodology of the 494 included scoping reviews relative to each of the steps recommended by the Joanna Briggs Institute guidance on scoping reviews.

# Appendix I. Description of key components of knowledge translation activities

| **Key components of studies that had integrated KT**   - Stakeholder involvement throughout length of the project in order to generate meaningful results for the knowledge users.   - Formation of advisory group and/or consultation with experts or representatives from relevant organizations.   - Help to shape the focus of review (i.e. research question), provide input on preliminary findings, ensure appropriate search strategy and eligibility criteria; suggest relevant materials, help to create data abstraction tools and interpret results; inform the discussion, help to prepare the final report and guide the dissemination strategy (i.e. KT activities, connecting with end-users).   **Key components of studies that had end-of-grant KT (or consultation phase as per the Arksey and O’Malley framework)**   - Consultation exercise with diverse stakeholder groups (e.g. organizations, key informants, knowledge users, content experts, people with direct experience, etc.) to explore perspectives about topic of interest.   - Participative conferences to present and discuss findings, gather feedback about application(s) or implications of the results.     - Opportunity to validate and enhance preliminary findings with end-users and plan how to use data to inform future recommendations or next steps; also to brainstorm knowledge gaps, or other issues.     - Achieve consensus on content and wording of key messages or other outcome measure(s), e.g. use of a modified Nominal Group Technique to identify research priorities.     - Analyse data thematically, or via a ‘framework’ technique (Ritchie & Spencer 1994)     - Gather feedback on the scope, structure, content, and layout of the final report.   - Primary data collection via interviews, focus groups or surveys to elicit expert views or opinions (to contrast with scoping review findings). - **Targeted dissemination activities/strategies**    - Presentations at key national conferences.   - Community outreach and partnerships with relevant organizations or groups in the community to identify strategies for information exchange and/or champions to help disseminate findings and/or recommendations.   - Creation of briefing notes (that highlight key messages, recommendations, or action items) or websites designed to share findings and engage end-users.   **Key components of studies that had both integrated and end-of-grant KT**   - Incorporated elements from the two types of KT described above, in addition to engaging with novel stakeholders for the end-of-grant or consultation piece (than those that participated throughout). |
| --- |
